# Marine Heatwave and Extreme Hypoxia Coincide With Rapid Coral Bleaching and Mortality on a Central Red Sea Coral Reef

**DOI:** 10.1002/ece3.74606

**Published:** 2026-11-04

**Authors:** Shannon G. Klein, Eleonora Re, Naira Pluma, Cassandra Roch, Carlos M. Duarte

**Affiliations:** ^1^ Marine Science Program, Biological and Environmental Science and Engineering Division (BESE) King Abdullah University of Science and Technology (KAUST) Thuwal Kingdom of Saudi Arabia

**Keywords:** acute heat stress, compound extreme events, coral tissue disintegration, coral tissue sloughing, low‐oxygen extremes

## Abstract

Coral bleaching and mortality are commonly associated with heat stress during marine heatwaves (MHWs), yet attributing these responses solely to heat stress may overlook the complexity of contemporary MHWs. Here, we document a severe MHW affecting a central Red Sea reef in mid‐2024 that coincided with repeated nighttime low‐oxygen events. By integrating satellite and in situ records of temperature, dissolved oxygen and wind, alongside repeated surveys of in situ nursery‐reared coral fragments, we document the temporal coincidence of these conditions with rapid coral bleaching and mortality. As temperatures approached and exceeded 33°C during the MHW peak, nighttime oxygen minima were extreme (down to 15.2–57.8 μmol O_2_ kg^−1^) and diel temperature variability diminished (down to 0.31°C–0.67°C), indicating a loss of nighttime thermal reprieves. Coincident responses of nursery corals were atypical, with transitions from healthy or bleached states to mortality occurring within 7–8 days. Bleaching and mortality also occurred across diverse coral species in nearby habitats after the MHW peak, with < 1% live coral cover documented eight months later. Sustained high temperatures, reduced vertical mixing and increased nighttime respiratory demand likely constrained oxygen availability, resulting in low‐oxygen extremes. We posit that similar compound MHW extreme events may have gone undetected in the Red Sea and elsewhere, potentially obscuring the contribution of co‐occurring low‐oxygen extremes to coral responses attributed to heat stress.

## Graphical abstract

We document a severe 2024 marine heatwave (MHW) on a central Red Sea reef that coincided with repeated nighttime low‐oxygen extremes and rapid coral bleaching and mortality. These responses included transitions from healthy or bleached states to mortality within 7–8 days, suggesting that some coral impacts during MHWs may reflect compound heat–oxygen stress rather than heat stress alone.

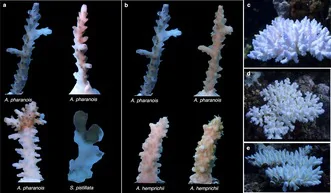

## 1 Introduction

Mass coral bleaching and mortality driven by climate change are threatening tropical coral reefs worldwide (Reimer et al. 2024; Duarte et al. 2025; Eakin et al. 2026). These responses are commonly associated with sustained periods of anomalously high sea surface temperatures (SSTs), known as marine heatwaves (MHWs) (Glynn 1993; Brown 1997; Hobday et al. 2016; Hughes et al. 2017). However, attributing such responses solely to heat stress may overlook the broader set of stressors that can co‐occur during contemporary MHWs (Cunio and Mercado‐Molina 2026; Klein et al. 2026). Recent global analyses reveal a marked increase in the co‐occurrence of MHWs with low‐oxygen and other biogeochemical extremes (Gruber et al. 2021; Li et al. 2024; Le Grix et al. 2025). These so‐called compound extreme events occur because unusually warm temperatures can reduce oxygen solubility, elevate metabolic oxygen demand and strengthen stratification, thereby restricting ventilation (Gruber et al. 2021; Deutsch et al. 2024; Li et al. 2024; Le Grix et al. 2025).

On tropical coral reefs, both long‐term warming and MHWs are expected to increase the frequency, duration and severity of low‐oxygen extremes (Lucey et al. 2023; Pezner et al. 2023). Several interacting processes render coral reefs particularly susceptible to low‐oxygen extremes during MHWs (Hughes et al. 2020). Heat stress can impair photosynthetic oxygen production, constraining daytime oxygen accumulation (Pezner et al. 2023; Parry, Klein, Steckbauer, et al. 2025), while elevated community respiration can push nighttime oxygen minima below critical biological thresholds (Hughes et al. 2020; Pezner et al. 2023). Reef geomorphology further modulates these dynamics, as enclosed or semi‐enclosed settings and unusually calm conditions can intensify warming‐induced stratification, reducing turbulent mixing and ventilation, which support oxygen resupply and heat loss (Guadayol et al. 2014; Andréfouët et al. 2015; Ruiz‐Jones and Palumbi 2015; Camp et al. 2017; Hughes et al. 2020). Such shifts in oxygen regimes are likely to amplify the impacts of MHWs on tropical coral reefs. Experimental evidence suggests that reduced oxygen availability exacerbates thermal stress in corals and other reef‐associated organisms (Alderdice et al. 2021, 2022; Gravinese et al. 2022; Lucey et al. 2022, 2023, 2024; Parry, Klein, Steckbauer, et al. 2025; Dhillon et al. 2026). However, the episodic nature of such compound extreme events, combined with limited high‐frequency in situ observations, constrains our ability to assess their prevalence and the extent to which they drive biological impacts in situ (Hobbs and McDonald 2010; Baird et al. 2018; Johnson et al. 2018, 2021; Lucey et al. 2024; Richards et al. 2024).

In April 2024, the National Oceanic and Atmospheric Administration (NOAA) and the International Coral Reef Initiative confirmed the Fourth Global Coral Bleaching Event (Reimer et al. 2024). Here, we report observations during a severe MHW in mid‐2024 that impacted a semi‐sheltered site on a mid‐shore reef in the central Red Sea. By integrating satellite records with in situ temperature, dissolved oxygen and wind records, together with repeated observations of in situ nursery‐reared coral fragments, we document the temporal coincidence of rapid coral bleaching and mortality with unusually warm, low‐wind conditions and low‐oxygen extremes. Observations of nearby reef habitats during and after the event, together with post‐event photo‐transects conducted months later, indicate severe bleaching, coral mortality and very low live coral cover at the study site.

## 2 Methods

### 2.1 Study Site and Coral Nursery Structure

This study was conducted at Al Fahal Reef, a mid‐shore reef in the central Red Sea near Thuwal, Saudi Arabia (22°18′11″ N, 38°57′53″ E). On 4 June 2024, a coral nursery structure was suspended mid‐water at ~9 m depth in a semi‐sheltered area at the northern tip of Al Fahal Reef (22°18′21.7″ N, 38°57′50″ E) as part of a separate experimental study (Figure S1) (Re and Duarte 2025). Briefly, we collected nine source colonies representing three coral species ( *Acropora pharaonis* , *Acropora hemprichii* and *Stylophora pistillata* ; three colonies per species) at depths of 2–10 m. Ten fragments (3–8 cm in length) were produced from each colony and randomly assigned to positions on the polyvinyl chloride (i.e., PVC) ‘tree’ nursery structure (total *n* = 90). The fragments were spaced at least 4 cm apart to minimise the potential interaction between fragment microenvironments. Although deployed for a separate experimental study (Re and Duarte 2025), the nursery structure provided a standardised framework for documenting in situ coral responses during the MHW.

Between 4 June and 15 August 2024, we monitored all nursery fragments at approximately weekly intervals for health status and cleaned the bases of all fragments. Fragments that remained visually unchanged from their initial deployment condition were classified as ‘unbleached’. Fragments exhibiting obvious signs of paling, partial bleaching or complete loss of pigmentation were classified as ‘bleached’. Fragments displaying bright host‐derived fluorescence, a response commonly associated with physiological stress in corals (Roth and Deheyn 2013; Bollati et al. 2020), were classified as ‘fluorescent’. Fragments that showed partial tissue disintegration (or ‘tissue sloughing’) but retained most living tissues were classified as ‘tissue disintegration’ (*sensu* Dhillon et al. 2026), whereas fragments exhibiting substantial tissue disintegration or algal colonisation consistent with fragment mortality were classified as ‘dead’.

### 2.2 In Situ Temperature and Dissolved Oxygen Measurements

On 17 July 2024, a miniDOT oxygen logger (PME) was attached to the base of the nursery structure at ~10 m depth to continuously record temperature (°C) and dissolved oxygen (mg L^−1^) at 5‐min intervals. The logger was positioned ~30 cm from the nearest coral fragment and ~2 m above the benthos, thereby capturing ambient mid‐water conditions. A two‐point calibration check was performed prior to deployment (using 100% air saturation and 0% oxygen (via nitrogen gas)), which confirmed no offset was required. The miniDOT oxygen sensor was cleaned at weekly intervals between 17 July and 15 August 2024. Raw dissolved oxygen time series were quality controlled by removing missing values and applying a rolling median–median absolute deviation de‐spiking procedure to flag and replace transient high‐ and low‐amplitude spikes using the ‘zoo’ package in R (v.4.3.0). Resulting dissolved oxygen concentrations (in mg L^−1^) were then converted to μmol kg^−1^ using temperature‐corrected solubility relationships assuming a constant salinity of 39 psu using the ‘respirometry’ package. Additional temperature and dissolved oxygen measurements from two earlier deployments at the same reef site (27 July–11 August 2023 and 13–28 August 2023) were collected using the same sensor configuration and sampling protocol (Figure S2). For these deployments, the loggers were attached to the reef substrate at ~10 m depth, with the sensor oriented outward into the water column. The in situ temperature and dissolved oxygen time series thus represent measurements from single loggers deployed at the study site.

### 2.3 Wind and Satellite SST Data

Wind speed data were obtained from a single SOFAR Spotter buoy deployed at Al Fahal, and daily mean wind speeds were calculated only for days with at least 12 valid records to avoid bias from incomplete daily coverage (Figure S3). Daily SST and sea surface temperature anomaly (SSTA) data were obtained from the NOAA Coral Reef Watch CoralTemp v3.1 product (5 km spatial resolution) for the study site for 1985–2024 (Skirving et al. 2020). SST and SSTA fields were extracted from the grid cell nearest to the study location. SSTA values represent the deviation of daily SST from the CoralTemp v3.1 climatological mean for the corresponding day of the year. We used daily SST data to calculate MHW characteristics for 2024, following ref. (Hobday et al. 2016), using the ‘heatwaveR’ package in R (v.4.3.0) (Schlegel and Smit 2018). A seasonally varying 90th‐percentile threshold, calculated from the 1985–2014 baseline period, defined events lasting at least five consecutive days above this percentile. The extracted event metrics included onset date, end date, duration, peak date, maximum intensity and mean intensity (Table S1).

### 2.4 Post‐Event Photo Transects

Post‐event surveys were conducted on 17 April 2025 to quantify benthic composition, including coral health status. The timing allowed the assessment of longer‐term outcomes and delayed coral impacts following the MHW. Three 25 m transects were first conducted in the semi‐sheltered reef area near the coral nursery deployment site (22°18′20.44″ N, 38°57′54.59″ E). Transects followed the natural reef contours and were spaced at least 5 m apart to ensure spatial independence.

Each transect was photographed at 1‐m intervals using an Olympus TG‐6 camera. Benthic composition was quantified as per cent cover using CPCe, following standard protocols (Kohler and Gill 2006). One quadrat image was excluded due to poor image quality; to maintain consistency, one quadrat was removed from each transect prior to analysis (*n* = 24 quadrat images per transect; Table S2). For each transect, 24 × 1 m^2^ photo quadrats were scored using 50 randomly generated points, yielding 1200 points per transect. We assigned each point to a benthic category. Live corals were identified to genus level whenever possible and recorded separately as either healthy or bleached (e.g., *Porites* sp. or *Porites* sp. bleached). When genus‐level identification was not possible, live corals were classified as ‘other hard coral’. When colonies were dead and algal growth obscured skeletal features, we classified points as ‘dead hard coral’. We assigned remaining points to non‐coral benthic categories, including turf algae, crustose coralline algae (CCA) or sponge.

### 2.5 Statistical Analyses

We used a Kaplan–Meier time‐to‐event analysis to compare the timing of impacts among species using the ‘survival’ package in R (v.4.3.0). For this analysis, fragments classified as ‘unbleached’ were considered unaffected, while those classified as ‘bleached’, ‘fluorescent’, ‘tissue disintegration’ or ‘dead’ were considered affected. The event was the first affected state observed. Fragments unbleached throughout (4 June–15 August 2024) were right‐censored at the last survey. Time was counted from 4 June to the first affected observation or the last survey. Kaplan–Meier curves were generated separately for each species, and differences among species were evaluated using a log‐rank test. The lack of independently replicated environmental time series during the event precluded formal statistical estimation of how environmental parameters affected in situ coral responses. Instead, we compared the timing of changes in environmental parameters with the timing of observed coral impacts and interpreted coral responses in relation to these conditions.

## 3 Results

### 3.1 Environmental Conditions During the 2024 MHW

SST anomalies (SSTA) at the study site were positive throughout 2024 (Figure 1). From mid‐June to August, SSTAs and SSTs exceeded the range observed in previous years (1985–2023), with only brief overlap with historical variability (Figure 1). Using the Hobday framework (Hobday et al. 2016), we identified a MHW that began on 3 June 2024 and persisted for 108 days (Table S1). This event reached a maximum intensity of 2.59°C on 12 August, corresponding to a peak SST of 33.7°C (Figure 1b,c; Table S1).

**FIGURE 1:**
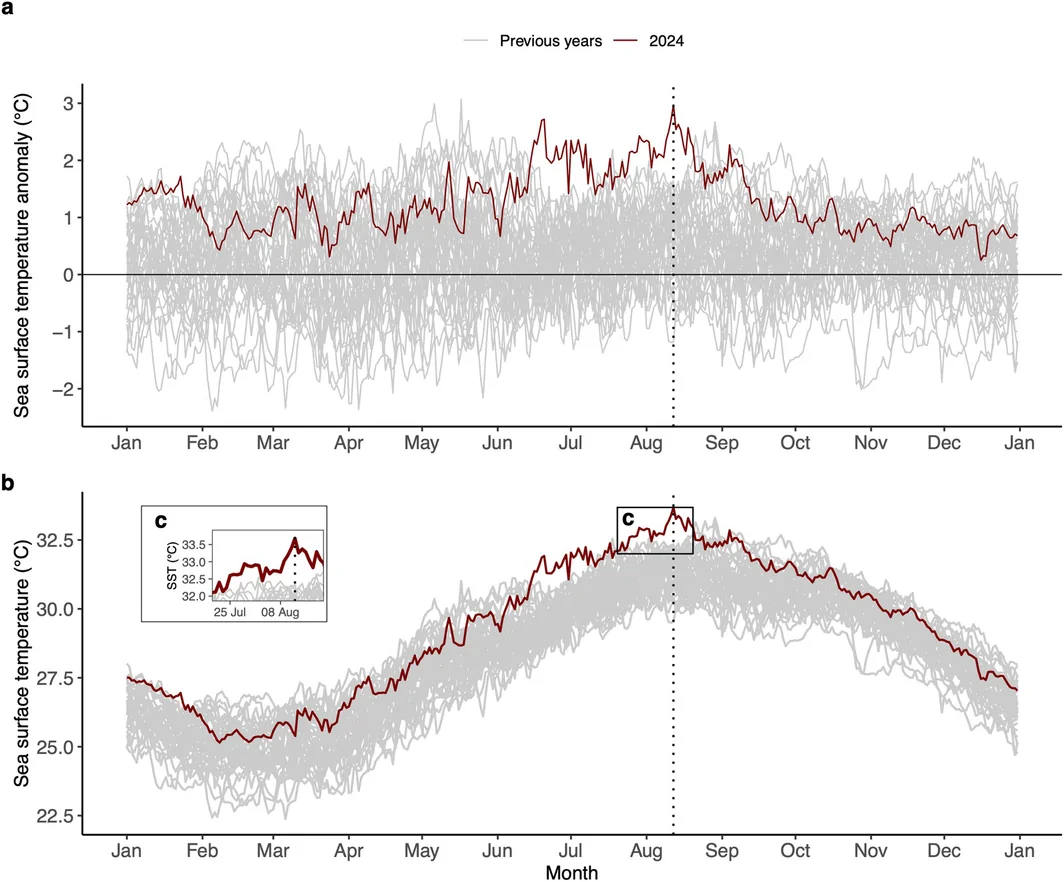
(a) Daily sea surface temperature anomaly (SSTA) and (b) mean sea surface temperature (SST) extracted for the study site (22.306° N, 38.964° E) from 1985 to 2024 from NOAA Coral Reef Watch CoralTemp v3.1 (5 × 5 km resolution) (Skirving et al. 2020). SSTA values represent the deviation of daily SST from the CoralTemp v3.1 climatological mean for the corresponding day of the year (Skirving et al. 2020). Grey lines represent years 1985–2023, and red indicates data for 2024. The boxed region within b indicates the period in which in situ temperature and dissolved oxygen were recorded (17 July–15 August) and is shown enlarged in the inset (c).

In situ temperatures at 10 m closely tracked satellite‐derived SSTs (Figures 1c and 2). Peak daily temperatures increased from 31.9°C on 18 July 2024 to 33.0°C–33.2°C by 29–31 July and remained elevated thereafter, reaching 33.9°C on 11 August, one day prior to the satellite‐derived peak (Figure 2). Consistent with records from summer 2023 (Figure S2), this warming coincided with a marked compression of diel temperature variability (daily max–min), declining from 0.9°C–2.2°C (18–27 July 2024) to 0.31°C–0.67°C (29 July–12 August 2024), indicating a loss of nighttime thermal reprieves (Figure 2). Wind speeds were low, with mean values ≤ 3 m s^−1^ on 11 of 13 observation days between 16 and 28 July (Figure S3), representing doldrum‐like conditions (Liu et al. 2012). Winds were slightly stronger thereafter but remained < 5 m s^−1^ between 1 and 15 August, and predominantly originated from the northwest to west, from which the study site is sheltered (Figures S1 and S3). Wind speed records from summer 2023 were more variable and primarily originated from the west, with mean values ≤ 3 m s^−1^ on 6 days between 16 and 28 July 2023, indicating fewer consecutive days of doldrum‐like conditions (Figure S4).

**FIGURE 2:**
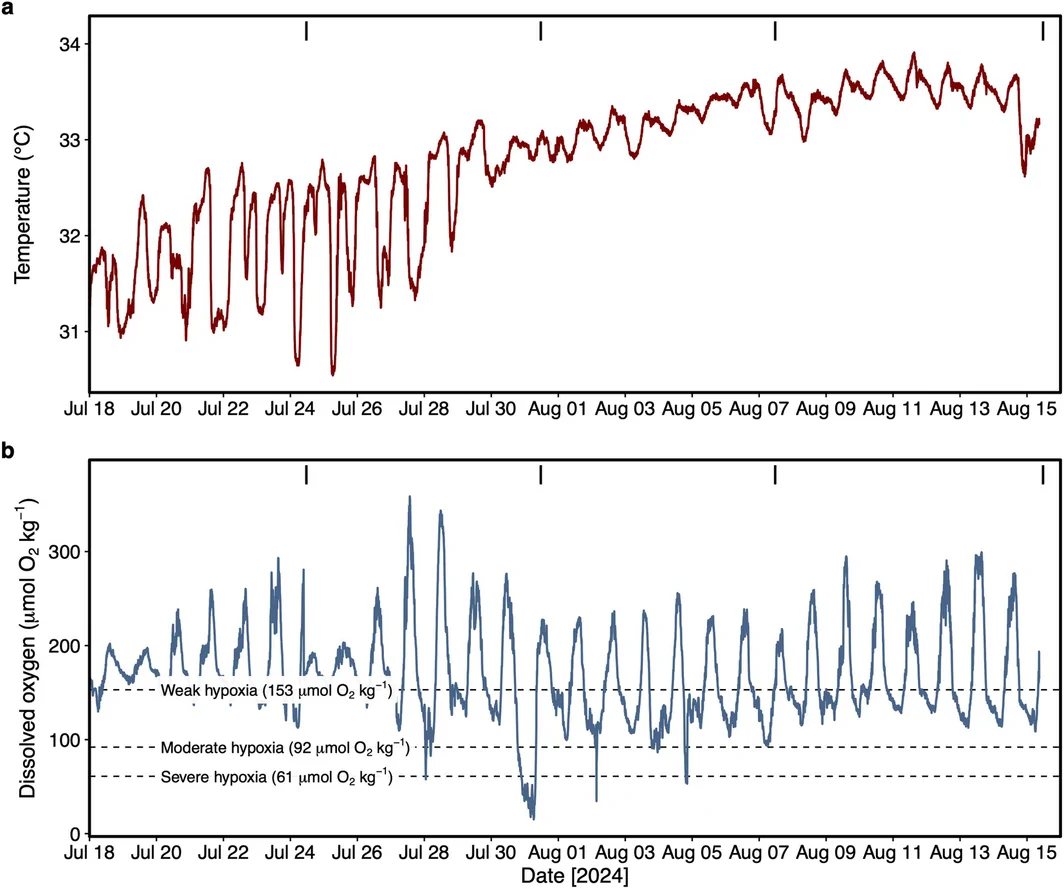
(a) In situ temperature and (b) dissolved oxygen time series measured at ~10 m depth at the study site during July–August 2024. Short vertical ticks denote the timing of in‐water nursery coral observations. Horizontal dashed lines in (b) indicate weak (153 μmol O_2_ kg^−1^), moderate (92 μmol O_2_ kg^−1^) and severe (61 μmol O_2_ kg^−1^) environmental hypoxia for tropical and subtropical reefs, according to ref. (Pezner et al. 2023).

Between 18 and 27 July 2024, pronounced diel temperature variability coincided with daytime maxima of 198–359 μmol O_2_ kg^−1^ and nighttime minima of 110–159 μmol O_2_ kg^−1^ (Figure 2b). These nighttime values approached and occasionally fell below the weak‐hypoxia threshold reported for tropical reefs (Pezner et al. 2023), consistent with oxygen dynamics during summer 2023 when temperatures similarly approached 33°C (Figure S2). When temperatures approached and exceeded 33°C under weak wind forcing, nighttime oxygen declined below the moderate– and severe–hypoxia thresholds (Figure 2) (Pezner et al. 2023). On 28 July, nighttime oxygen dropped to 57.8 μmol O_2_ kg^−1^, indicating severe hypoxia and then declined further to 34.7 and 15.2 μmol O_2_ kg^−1^ on 30 and 31 July, respectively, while daytime oxygen remained high (> 220 μmol O_2_ kg^−1^; > 124% air saturation). Nighttime oxygen minima partially recovered to ~93 μmol O_2_ kg^−1^ on 7 August and remained above the moderate‐hypoxia threshold thereafter (Figure 2b), coinciding with slightly greater wind speeds relative to late July (Figure S3).

### 3.2 Nursery Coral Responses During Peak MHW Conditions

Biological impacts to nursery coral fragments were first evident in *A. hemprichii* and *A. pharaonis* on 24 July 2024, while *S. pistillata* remained unaffected (Figure 3). In the week prior (18–24 July), daily maximum temperature averaged 32.47°C ± 0.35°C (mean ± SD across days), diel temperature range averaged 1.52°C ± 0.39°C and daily mean wind speed averaged 2.64 ± 0.53 m s^−1^ (Figure 2, Table 1). Overnight oxygen levels fell below the weak‐hypoxia threshold on 6 of 7 nights but did not reach moderate or severe hypoxia thresholds (Table 1).

**FIGURE 3:**
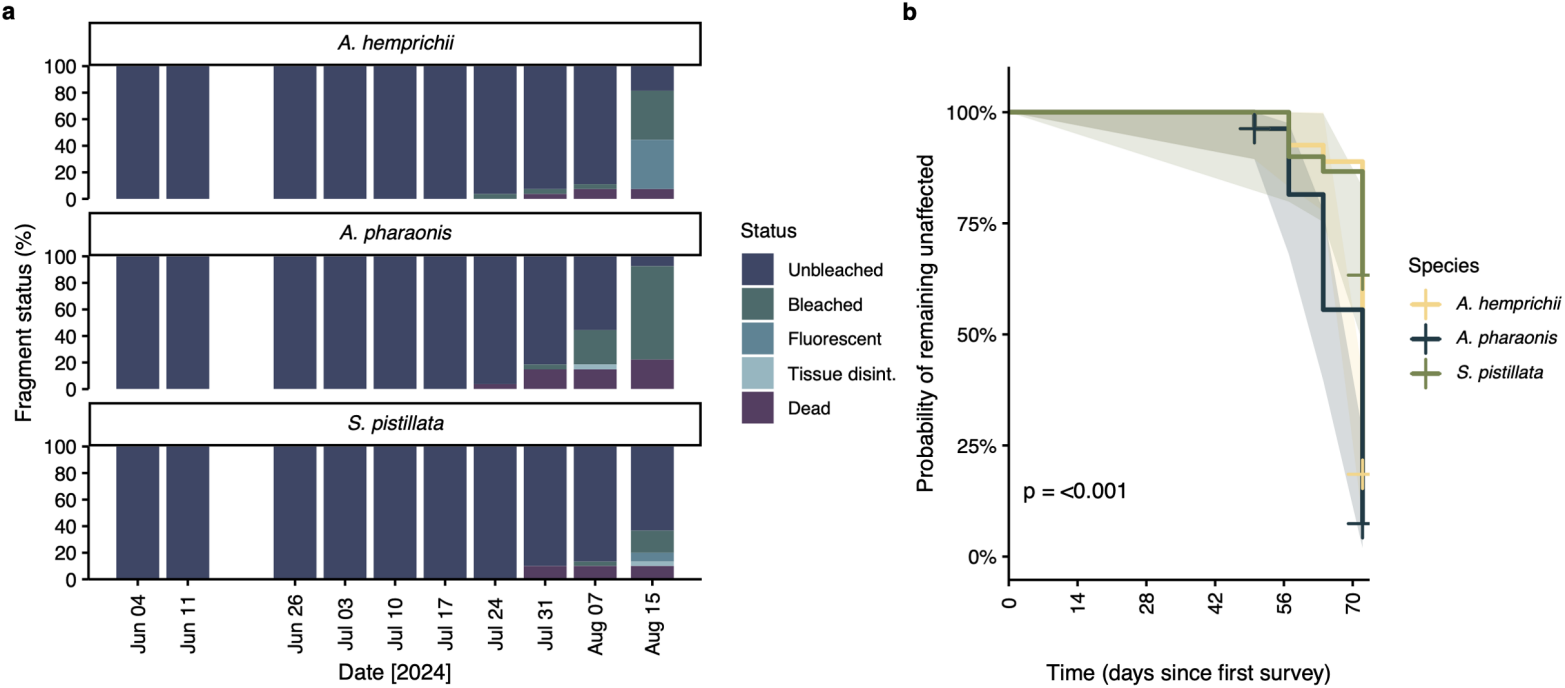
(a) Temporal changes in the status of in situ nursery‐reared coral fragments for each species, showing the proportion of colonies classified as unbleached, bleached, fluorescent, tissue disintegration (tissue disint.) or dead at each survey. (b) Kaplan–Meier estimates of the probability of remaining unaffected over time, where unaffected is defined as unbleached and events correspond to the first observation of an affected state (bleached, fluorescent, tissue disintegration or dead). Shaded regions indicate 95% confidence intervals; differences among species were assessed using a log‐rank test.

**TABLE 1:** Environmental conditions during successive coral survey intervals between 18 July and 15 August 2024.

Environmental variable	18–24 Jul	25–31 Jul	1–7 Aug	8–15 Aug
Daily maximum temperature (mean ± 1SD, °C)	32.47 ± 0.35	32.96 ± 0.19	33.44 ± 0.17	33.68 ± 0.22
Diel temperature range (mean ± 1SD, °C)	1.52 ± 0.39	1.13 ± 0.66	0.43 ± 0.11	0.54 ± 0.22
Overnight minimum O_2_ (range, μmol O_2_ kg^−1^)	112.7–159.0	15.2–152.0	34.6–106.8	108.8–128.4
Number of nights < 153 μmol O_2_ kg^−1^	6 of 7	7 of 7	7 of 7	8 of 8
Number of nights < 92 μmol O_2_ kg^−1^	0 of 7	2 of 7	3 of 7	0 of 8
Number of nights < 61 μmol O_2_ kg^−1^	0 of 7	2 of 7	2 of 7	0 of 8
Cumulative hours < 153 μmol O_2_ kg^−1^ (h)	35.0	63.0	102.0	97.6
Cumulative hours < 92 μmol O_2_ kg^−1^ (h)	0	14.3	3.6	0
Cumulative hours < 61 μmol O_2_ kg^−1^ (h)	0	10.4	0.8	0
Daily mean wind speed (mean ± 1SD, m s^−1^)	2.64 ± 0.53	2.23 ± 0.43	3.63 ± 0.76	4.16 ± 0.84

*Note:* Daily maximum temperature, diel temperature range and daily mean wind speed are presented as mean ± 1 standard deviation (SD) across days within each interval. Overnight minimum dissolved oxygen (O_2_) is presented as the range of minima recorded between 19:00 and 06:59, with each overnight period assigned to the date on which it ended. The number of nights below weak (< 153 μmol O_2_ kg^−1^), moderate (< 92 μmol O_2_ kg^−1^) and severe (< 61 μmol O_2_ kg^−1^) hypoxia thresholds are reported as the number of affected nights out of the total observed within each interval. Cumulative hours below each threshold are calculated across the full 24‐h record. Hypoxia thresholds are nested, such that time below the lower thresholds is also included in the higher‐threshold totals.

Nursery coral responses then progressed rapidly through late July and early August 2024 (Figures 3 and Figure 4). Specifically, between 25 and 31 July, daily maximum temperature averaged 32.96°C ± 0.19°C, while daily mean wind speed averaged 2.23 ± 0.43 m s^−1^. Oxygen fell below the moderate– and severe–hypoxia thresholds on 2 of 7 nights, with cumulative periods of 14.3 and 10.4 h below the respective thresholds (Table 1). In some cases, rapid transitions from apparently healthy coral fragments to fragment mortality occurred within 7–8 days (Figures 3 and 4b). For example, by 31 July, *S. pistillata* fragments exhibited 10% mortality, with no impacts observed the week prior (Figure 3). By this same date, *A. pharaonis* exhibited 3.7% bleaching and 14.8% mortality, while *A. hemprichii* exhibited 3.7% bleaching and 3.7% mortality (Figure 3).

**FIGURE 4:**
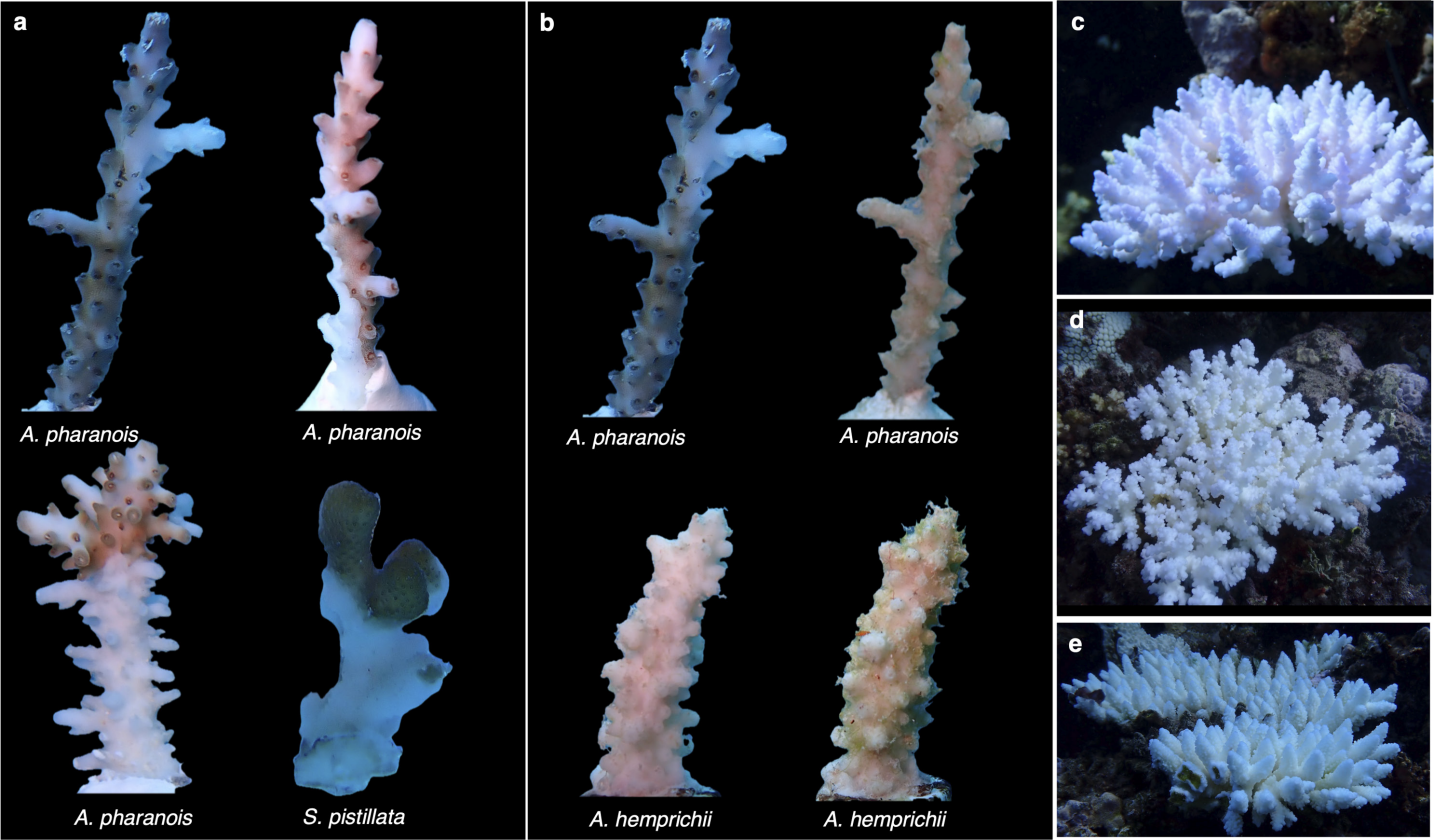
(a) Tissue disintegration in *Acropora pharaonis* and *Stylophora pistillata* . Top left and right ( *A. pharaonis* ): Partial tissue disintegration, with exposed skeleton while most tissue remains (classified as ‘tissue disintegration’; see Methods). Bottom left ( *A. pharaonis* ): Extensive tissue disintegration with widespread skeleton exposure, classified as ‘dead’ (see Methods). Bottom right ( *S. pistillata* ): Extensive tissue disintegration with widespread skeleton exposure, also classified as ‘dead’. (b) Progression over 8 days. Top left: *A. pharaonis* on 31 July 2024 exhibiting partial tissue disintegration (left; also, in a), followed 8 days later by mortality with algal colonisation (top right). Bottom: *Acropora hemprichii* exhibiting bleaching (complete loss of pigmentation with mucus presence; left), followed 8 days later (7 August 2024) by mortality with algal colonisation (right). (c–e) (21 August 2024) show a bleached *Acropora* sp. colony, a bleached *Pocillopora* sp. colony showing early signs of algal colonisation and a bleached *Acropora* sp. colony, with signs of early algal colonisation, respectively.

Between 1 and 7 August 2024, the daily maximum temperature increased further to ~33.4°C and the diel temperature range compressed to ~0.4°C (Table 1). Moderate and severe hypoxia occurred on 3 and 2 of 7 nights, respectively, while mean wind speed slightly increased to ~3.6 m s^−1^ (Table 1). Overall, the probability of coral fragments remaining unaffected declined over time and differed among species (Figure 3b, log‐rank test, *p* < 0.001), with divergence among species emerging over this interval. *A. pharaonis* showed the steepest decline, while *S. pistillata* retained the highest probability of remaining unaffected (Figure 3b).

During the final observation interval (8–15 August 2024), daily maximum temperature remained elevated at ~33.7°C and diel temperature range remained compressed at ~0.5°C, while mean wind speed increased further to ~4.2 m s^−1^. Overnight oxygen minima remained below the weak–hypoxia threshold on all 8 nights, but levels did not decline to the moderate and severe hypoxia thresholds (Table 1). By 15 August, only 29% of all fragments remained unaffected (Figure 3). Impacts were most pronounced in *A. pharaonis* (70.4% bleached, 22.2% dead), followed by *A. hemprichii* (37.0% bleached, 37.0% fluorescent, 7.4% dead). In contrast, *S. pistillata* showed lower bleaching and fluorescence but comparable mortality, with some instances of tissue disintegration (16.7% bleached, 6.7% fluorescent, 3.3% tissue disintegration, 10% dead) (Figure 3). Tissue disintegration occurred in all species and, when observed, was invariably followed by mortality within 7–8 days (Figure 4). During the recorded period spanning the MHW peak (18 July–15 August), cumulative exposure below the weak–, moderate– and severe–hypoxia thresholds totalled 297.6, 17.9 and 11.2 h, respectively (Table 1).

Following the peak of the MHW (21 August 2024), bleaching and mortality were documented across diverse coral species in nearby reef habitats, with few coral taxa remaining unaffected (Figure 4, Table S3). Some surviving bleached colonies showed signs of partial tissue mortality (e.g., *Stylophora* sp., *Pocillopora* sp., *Galaxea fascicularis* ), with affected tissues colonised by algae (Table S3). In other cases, dead colonies were overgrown by algae (e.g., *Acropora* sp.) (Table S3). Post‐event surveys conducted in April 2025 in the same nearby habitats at 7 m depth indicated extremely low live coral cover (~0.1%, comprising only healthy and bleached *Porites* sp.), with a high proportion of dead coral (31.7%) (Figure S5). Pre‐event photo‐transects of the study site were not available for comparison.

## 4 Discussion

Here, we show that low wind conditions during the peak of a severe MHW coincided with nighttime low‐oxygen extremes, consistent with a compound extreme event (Gruber et al. 2021; Lucey et al. 2022; Li et al. 2024; Le Grix et al. 2025). These observations align with projections that MHWs increase the occurrence and extent of low‐oxygen extremes on reefs (Lucey et al. 2023; Pezner et al. 2023), and with prior work showing that elevated temperatures alter reef oxygen regimes (Hobbs and McDonald 2010; de Verneil et al. 2021). The MHW event lasted 108 days and was the most intense recorded at the study site since those satellite records began in 1985. We hypothesise that elevated temperatures, weak wind forcing and direction and the site's semi‐sheltered geomorphology constrained vertical mixing and oxygen resupply. Combined with elevated community respiration, these conditions probably intensified nighttime oxygen drawdown, contributing to the low‐oxygen extremes observed. While sustained low‐oxygen events on reefs have been linked to anomalous heat and excess nutrients or organic matter (Johnson et al. 2018, 2021; Richards et al. 2024), only one study has documented similarly rapid and atypical coral responses under unusually warm and calm conditions without nutrient or organic matter enrichment (Baird et al. 2018). In that case, low oxygen was hypothesised to contribute to rapid coral bleaching and mortality (within 2 weeks), although oxygen was not measured.

Nursery coral responses during this event differ from descriptions of typical in situ thermal bleaching, in which mortality usually occurs over several weeks to months following bleaching (Glynn 1984; Baird and Marshall 2002; Baird et al. 2018). Here, transitions from healthy or bleached states to fragment death sometimes occurred within 7–8 days, and partial tissue disintegration was consistently followed by fragment mortality within the same timeframe. In some cases, coral tissue disintegration occurred without prior paling (see Figure 4) (Hoegh‐Guldberg et al. 2023; Dhillon et al. 2026), consistent with reports from southern Japan (Baird et al. 2018), the Caribbean and the eastern tropical Pacific (Hoegh‐Guldberg et al. 2023). In southern Japan, similar patterns were linked to abnormally warm, calm conditions, with low oxygen hypothesised as a driver (Baird et al. 2018), whereas reports from the Caribbean and eastern tropical Pacific were attributed to acute heat stress (Hoegh‐Guldberg et al. 2023). While observations of nursery corals were consistent with those of established colonies in nearby habitats following the MHW peak, nursery fragments were relatively small (3–8 cm) and fragment size may have influenced the timing and extent of their responses. Smaller colonies can exhibit greater susceptibility to bleaching and whole‐colony mortality, potentially reflecting differences in colony‐wide energetic resources, although these relationships vary among taxa and growth forms (Oren et al. 2001; Madin et al. 2014; Johnston et al. 2020). In contrast, mass transfer of dissolved gases and other solutes per unit surface area can be more rapid in smaller colonies, potentially improving exchange with the surrounding seawater during thermal stress (Nakamura and van Woesik 2001). This may be particularly relevant to the event reported here, as enhanced mass transfer could facilitate oxygen uptake from the surrounding seawater when metabolic oxygen demand increases under heat stress and oxygen availability becomes limiting (Parry, Klein, Steckbauer, et al. 2025; Dhillon et al. 2026).

The atypical coral responses observed here differ from those reported at reefs near the study site in summer 2023. There, temperatures reached ~33°C and bleaching persisted from September to November, followed by signs of recovery in February 2024 (Mayfield 2025), consistent with the typical trajectory of thermal bleaching. By contrast, bleaching and mortality of nursery corals were followed by extremely low coral cover in nearby habitats eight months later (~0.1% in April 2025, including bleached colonies), consistent with limited recovery at the study site, though estimates are based on limited spatial sampling (*n* = 3 transects covering 72 m^2^). During the same 2024 MHW, surveys at a nearby exposed site on Al Fahal similarly documented severe bleaching (74%–95% bleached coral cover) and subsequent coral cover declines (Dunn et al. 2026), though the fine‐scale timing of bleaching and mortality and associated oxygen dynamics were not resolved. Live coral cover at that exposed site declined from 28%–50% in August–September 2024 to 9%–16% in January 2025, indicating substantial post‐bleaching declines that were also consistent with surveys of nearby reefs (Mayfield 2025; Dunn et al. 2026).

The low‐oxygen extremes reported here surpassed the defined thresholds for weak, moderate and severe hypoxia on tropical reefs (153, 92 and 61 μmol O_2_ kg^−1^, respectively) (Pezner et al. 2023). The extremes reported during this event are comparable to, and sometimes lower than, oxygen concentrations used in experimental studies of combined thermal and low‐oxygen stress in corals (Alderdice et al. 2021, 2022; Parry, Klein, Steckbauer, et al. 2025). Such studies, including work from the central Red Sea, indicate that low‐oxygen exposure increases coral vulnerability to heat stress (Alderdice et al. 2021, 2022; Lucey et al. 2024; Parry, Klein, Steckbauer, et al. 2025; Johnson et al. 2026), likely because higher temperatures raise holobiont oxygen demand beyond available supply (Alderdice et al. 2022; Parry, Klein, and Duarte 2025; Parry, Klein, Steckbauer, et al. 2025; Dhillon et al. 2026). Recent work further shows that, under low‐flow conditions, this imbalance can drive oxygen deprivation within coral holobiont tissues even when oxygen availability in the surrounding seawater remains high, because oxygen transport cannot keep pace with metabolic demand (Dhillon et al. 2026). The combination of high temperatures and transient low‐oxygen extremes observed here may therefore have further exacerbated this supply–demand imbalance, contributing to the rapid and atypical coral responses during this event. Evidence from other reef organisms further supports this possibility, as combined temperature and transient low‐oxygen extremes have been shown to impair performance in both tropical sea urchins and brittle stars (Lucey et al. 2020, 2023). Despite growing recognition that nighttime oxygen extremes during MHWs can reach critical biological thresholds (Hughes et al. 2020; Pezner et al. 2023; Parry, Klein, Steckbauer, et al. 2025), there are few empirical tests of how dynamic oxygen regimes interact with sustained heat stress to affect coral responses, warranting further investigation (Hughes et al. 2020; Lucey et al. 2024).

Responses varied across nursery species, with *S. pistillata* showing the highest likelihood of remaining unaffected during the event. This aligns with observations of the 2020 bleaching event in the central Red Sea, where *Stylophora* sp. exhibited relatively high resistance and recovery, whereas *Acropora* spp. were among the taxa most severely impacted (Dunn et al. 2025). In contrast, an experimental study examining species‐specific responses of Al Fahal corals to acute heat stress reported a higher thermal threshold for *A. hemprichii* than for *S. pistillata* (Evensen et al. 2022). This discrepancy may reflect differences between short‐term laboratory experiments and prolonged in situ exposure, and/or the influence of environmental factors other than temperature. Evidence for species‐specific responses to combined heat and low oxygen is limited in the Red Sea, but one study examining *A. hemprichii* and *S. pistillata* found that low‐oxygen conditions (~1.2 mg O_2_ L^−1^) reduced the thermal threshold of *A. hemprichii* relative to those exposed to higher oxygen levels, whereas *S. pistillata* maintained similar thresholds across the different oxygen levels tested (Parry, Klein, Steckbauer, et al. 2025). Species‐level traits such as morphology may also contribute to variation in responses among these taxa. For instance, *A. hemprichii* has an open‐branching morphology (Wallace et al. 2012) and *A. pharaonis* forms large horizontal tables (Wallace et al. 2012), whereas *S. pistillata* forms more compact, spherical colonies with thick, rounded branches (Loya 1976). The two *Acropora* species also have higher estimated levels of colony integration than *S. pistillata* (Re and Duarte 2025). Such differences in colony architecture can alter within‐colony flow and mass transfer, with branch spacing and morphology influencing exchange between coral surfaces and the surrounding seawater (Reidenbach et al. 2006), potentially contributing to differences in stress susceptibility among species.

More broadly, other possible drivers could have contributed to the rapid and severe impacts observed across nursery corals and nearby habitats. First, as in summer 2023, diel temperature variability was markedly compressed as temperatures reached and exceeded 33°C, resulting in a lack of nighttime thermal reprieves. In situ and experimental investigations show that exposure to greater thermal variability typically enhances coral thermal tolerance (Schoepf et al. 2015, 2022; Safaie et al. 2018). Although not tested in corals, one study demonstrated that the absence of nighttime reprieves reduced the maximum temperatures a cnidarian holobiont model (*Cassiopea* sp.) could withstand (Klein et al. 2019). The loss of nighttime thermal reprieves observed here could therefore have limited recovery from daytime heat stress, potentially reducing tolerance to sustained temperature maxima.

Second, severe reductions in oxygen almost always coincide with acidification because community respiration consumes oxygen and simultaneously produces carbon dioxide, increasing dissolved inorganic carbon and lowering pH through the formation and dissociation of carbonic acid (Klein et al. 2020; Lucey et al. 2020). Such fluctuations in carbonate chemistry are well documented on reefs in the Red Sea and elsewhere, and correlate with changes in oxygen levels, with both pH and oxygen increasing during the day and decreasing at night (Smith et al. 2013; Lucey et al. 2020; Ziegler et al. 2021; Cryer et al. 2023). Reports of acute deoxygenation events on reefs further corroborate this coupling, showing concurrent deoxygenation and acidification (down to pH 7.46 in the Caribbean, Johnson et al. 2021). Experimental and field studies in non‐coral reef taxa indicate that short‐lived extremes in temperature and oxygen can have rapid biological impacts (Lucey et al. 2020, 2022), though co‐occurring pH reductions had minimal effects on sea urchins over short timescales (hours) (Lucey et al. 2020). In corals, however, interactions among warming, oxygen and acidification remain poorly resolved. One recent multi‐stressor study showed that hypoxia synergistically amplified the effects of heat stress on corals, whereas the responses to combined hypoxia and acidification were largely additive (Johnson et al. 2026). Although this study did not characterise fluctuations in the seawater carbonate system, parallel acidity extremes likely occurred and may have contributed to the observed impacts.

In summary, we show that atypical coral bleaching and mortality of nursery coral fragments coincided with an intense MHW, weak wind forcing and low‐oxygen extremes at the study site. Bleaching and tissue disintegration were also observed in nearby habitats across diverse coral species at the MHW peak and shortly thereafter, and post‐event surveys showed high proportions of dead coral and very low coral cover (~0.1%). High‐frequency oxygen records were ultimately confined to a single sensor at the study site, and these extremes may have been restricted to this location or to reefs with similar geomorphology and wind exposure. Because all nursery fragments shared the same environmental time series, these observations cannot resolve the relative contributions of temperature, low oxygen, wind and other potentially contributing factors. As MHWs intensify, such compound extreme events are expected to become more frequent (Le Grix et al. 2025), yet their ecological effects on reefs remain poorly resolved in both observational and empirical studies and current alert systems do not specifically address the risk of concurrent extreme temperatures and low oxygen. These observations raise the question of whether other similar events may have gone undetected in the Red Sea and elsewhere, potentially leading to the misattribution of coral bleaching and mortality solely to heat stress. As the ecological effects of concurrent heat and low‐oxygen extremes become better resolved, prolonged periods of low wind during MHWs may provide a readily available indicator of conditions conducive to reduced mixing and oxygen resupply, offering a path towards modifying early warning systems. More broadly, hydrodynamic forcing may influence coral responses across a range of conditions, with high wave energy also associated with increased bleaching during MHWs (Cunio and Mercado‐Molina 2026). While the role of hydrodynamic conditions in modulating coral heat stress is well recognised (e.g., Liu et al. 2012), oxygen and carbonate chemistry remain rarely monitored during MHWs.

## Author Contributions

**Shannon G. Klein:** conceptualization (lead), data curation (lead), formal analysis (lead), investigation (supporting), methodology (equal), project administration (equal), software (lead), supervision (equal), validation (equal), visualization (lead), writing – original draft (lead), writing – review and editing (equal). **Eleonora Re:** data curation (supporting), investigation (lead), methodology (equal), validation (equal), writing – review and editing (equal). **Naira Pluma:** data curation (supporting), investigation (supporting), methodology (supporting), visualization (supporting), writing – review and editing (equal). **Cassandra Roch:** formal analysis (supporting), investigation (supporting), methodology (supporting), validation (supporting), writing – review and editing (equal). **Carlos M. Duarte:** conceptualization (supporting), funding acquisition (lead), project administration (equal), resources (lead), supervision (equal), writing – review and editing (equal).

## Funding

This work was supported by the King Abdullah University of Science and Technology (KAUST) Office of Sponsored Research (OSR) through baseline funding awarded to CMD (Grant BAS/1/1071‐01‐01).

## Conflicts of Interest

The authors declare no conflicts of interest.

## Supporting information


**Figure S1:** (a) Map of the Red Sea highlighting the region near Thuwal, Saudi Arabia. (b) Satellite image of reefs offshore Thuwal, Saudi Arabia, with the boxed area indicating the study site and the extent of panel c. (c) High‐resolution satellite image of the northern tip of Al Fahal Reef showing the location of the study site and its semi‐enclosed geomorphology taken from Google Earth Pro. Scale bars denote distance.
**Figure S2:** (a) In situ temperature and (b) dissolved oxygen time series measured at ~10 m depth at the study site during July–August 2023. Horizontal dashed lines in b indicate weak (153 μmol O₂ kg^−1^), moderate (92 μmol O₂ kg^−1^) and severe (61 μmol O₂ kg^−1^) environmental oxygen depletion, according to ref. (Pezner et al. 2023). Note that the *y*‐axis scale for dissolved oxygen differs from that in Figure 2b of the main text, and the data gap reflects sensor calibration and redeployment. The period 27 July–15 August overlaps with the dates shown in Figure 2 for 2024.
**Figure S3:** (a) Wind speed (m/s) recorded by the SOFAR Spotter buoy at Al Fahal Reef in the central Red Sea from 30 June to 31 August 2024. Raw wind‐speed measurements are shown in grey, and the daily mean wind speed is overlaid in green. The shaded period, 27 July–15 August, overlaps with the dates shown in Figure 2. Daily means are not shown for days with fewer than *n* = 12 records to avoid bias due to incomplete daily coverage. Panels b and c show wind‐direction rose plots for the full record (30 June to 31 August 2024) and for the highlighted period (18 July to 15 August 2024), respectively.
**Figure S4:** (a) Wind speed (m/s) recorded by the SOFAR Spotter buoy at Al Fahal Reef in the central Red Sea from 30 June to 31 August 2023. Raw wind‐speed measurements are shown in grey, and the daily mean wind speed is overlaid in green. Panel b shows wind‐direction rose plots for the full record (30 June to 31 August 2023). *y*‐axis range in panel a differs from Figure S3a.
**Figure S5:** (a) Mean percent benthic cover across the three transects in April 2025 on nearby reef habitats, based on CPCe analysis of photo quadrats. (b) Mean percent cover (%) for each transect separately. (a, b) Bars are stacked by benthic category, and the *y*‐axis break improves visibility of low cover categories.
**Table S1:** Detected marine heatwaves in 2024 at the study site and their characteristics.
**Table S2:** Timing, location and depth of photo transects surveyed at the study site near the nursery structure location on Al Fahal Reef during post‐event surveys.
**Table S3:** Images of coral reef taxa in habitats near the coral nursery location after the MHW peak (21 August 2024), showing bleaching and mortality (as evidenced by algal colonisation).

## Data Availability

Daily SST and SSTA data are available from the NOAA Coral Reef Watch CoralTemp v3.1 product. All other data generated by this study are available on Dryad at https://doi.org/10.5061/dryad.k3j9kd5qh.
