# Supplementary material for: Marine Heatwave and Extreme Hypoxia Coincide With Rapid Coral Bleaching and Mortality on a Central Red Sea Coral Reef
Source: Ecol Evol. 2026 Nov 4;16(11):e74606. doi: 10.1002/ece3.74606 (PMC13736162; doi:10.1002/ece3.74606)
Supplement: Supplementary file 1 [file ECE3-16-e74606-s001.pdf]

Supplemental Information for

Marine heatwave and low-oxygen extremes coincide with coral bleaching and mortality on a central Red Sea coral reef

Shannon G. Klein*, Eleonora Re, Naira Pluma, Cassandra Roch and Carlos M. Duarte

Marine Science Program, Biological and Environmental Science and Engineering Division (BESE), King Abdullah University of Science and Technology (KAUST), Thuwal 23955-6900, Kingdom of Saudi Arabia

*Shannon G. Klein

Email: shannon.klein@kaust.edu.sa

This PDF file includes:

Figures S1 to S5

Tables S1 to S3

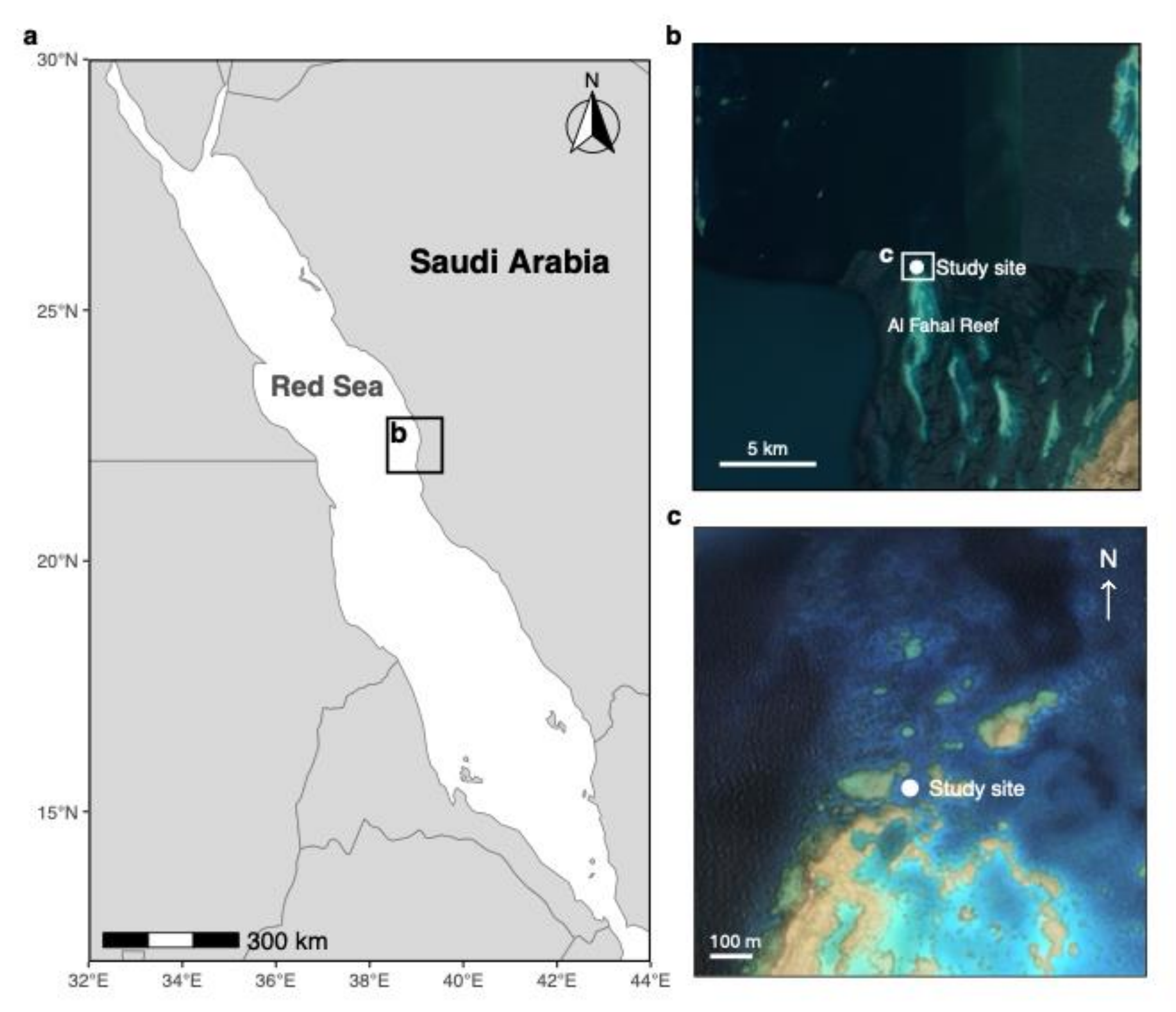

Figure S1 a, Map of the Red Sea highlighting the region near Thuwal, Saudi Arabia. **b,** Satellite image of reefs offshore Thuwal, Saudi Arabia, with the boxed area indicating the study site and the extent of panel c. **c,** High-resolution satellite image of the northern tip of Al Fahal Reef showing the location of the study site and its semi-enclosed geomorphology taken from Google Earth Pro. Scale bars denote distance.

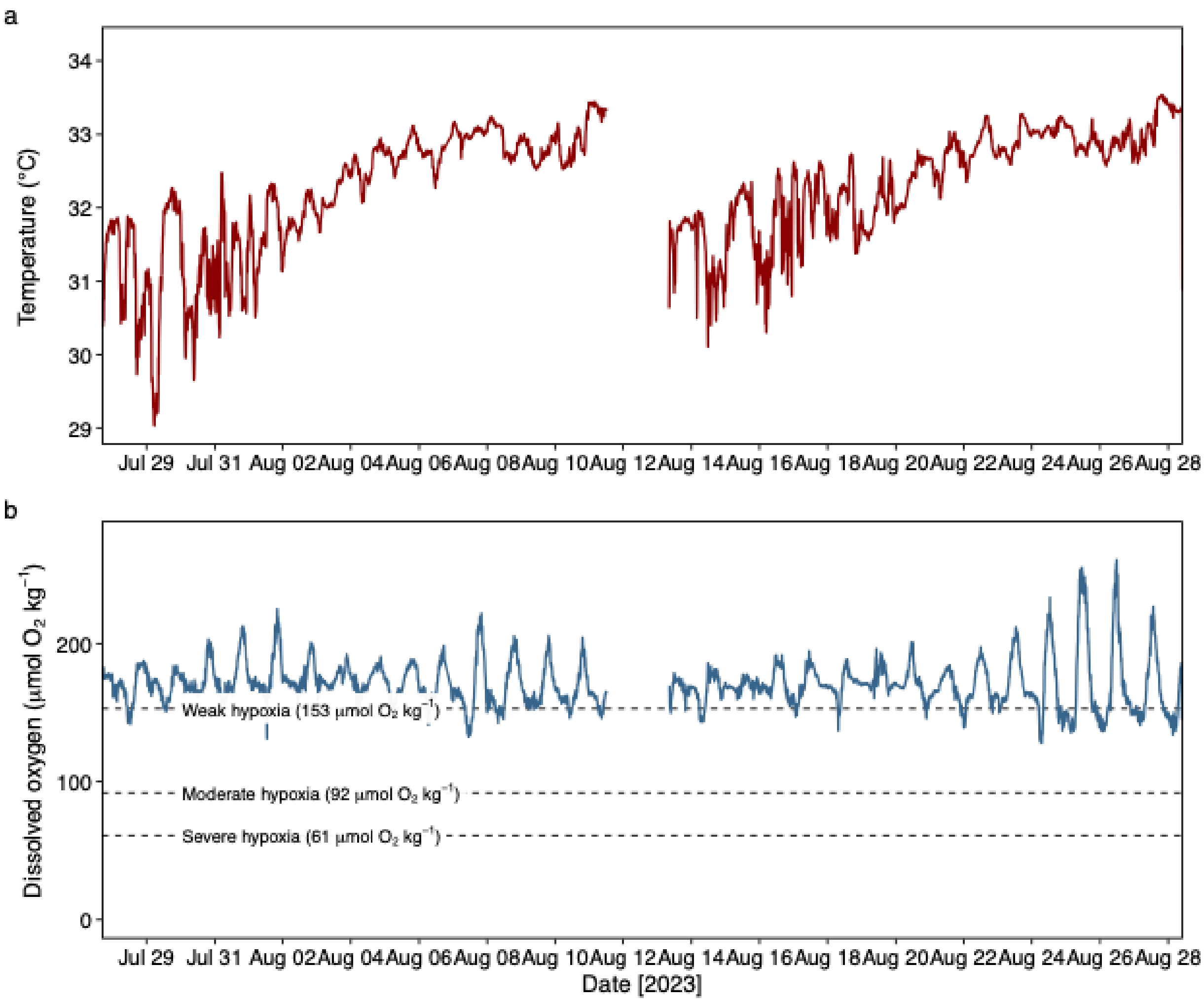

Figure S2 a, In situ temperature and **b**, dissolved oxygen time series measured at ~10 m depth at the study site during July–August 2023. Horizontal dashed lines in b indicate weak (153 μmol O_2 kg^{-1}), moderate (92 μmol O_2 kg^{-1}), and severe (61 μmol O_2 kg^{-1}) environmental oxygen depletion, according to ref. (Pezner et al. 2023). Note that the y-axis scale for dissolved oxygen differs from that in Fig. 2b of the main text, and the data gap reflects sensor calibration and redeployment. The period 27 July–15 August overlaps with the dates shown in Fig. 2 for 2024.

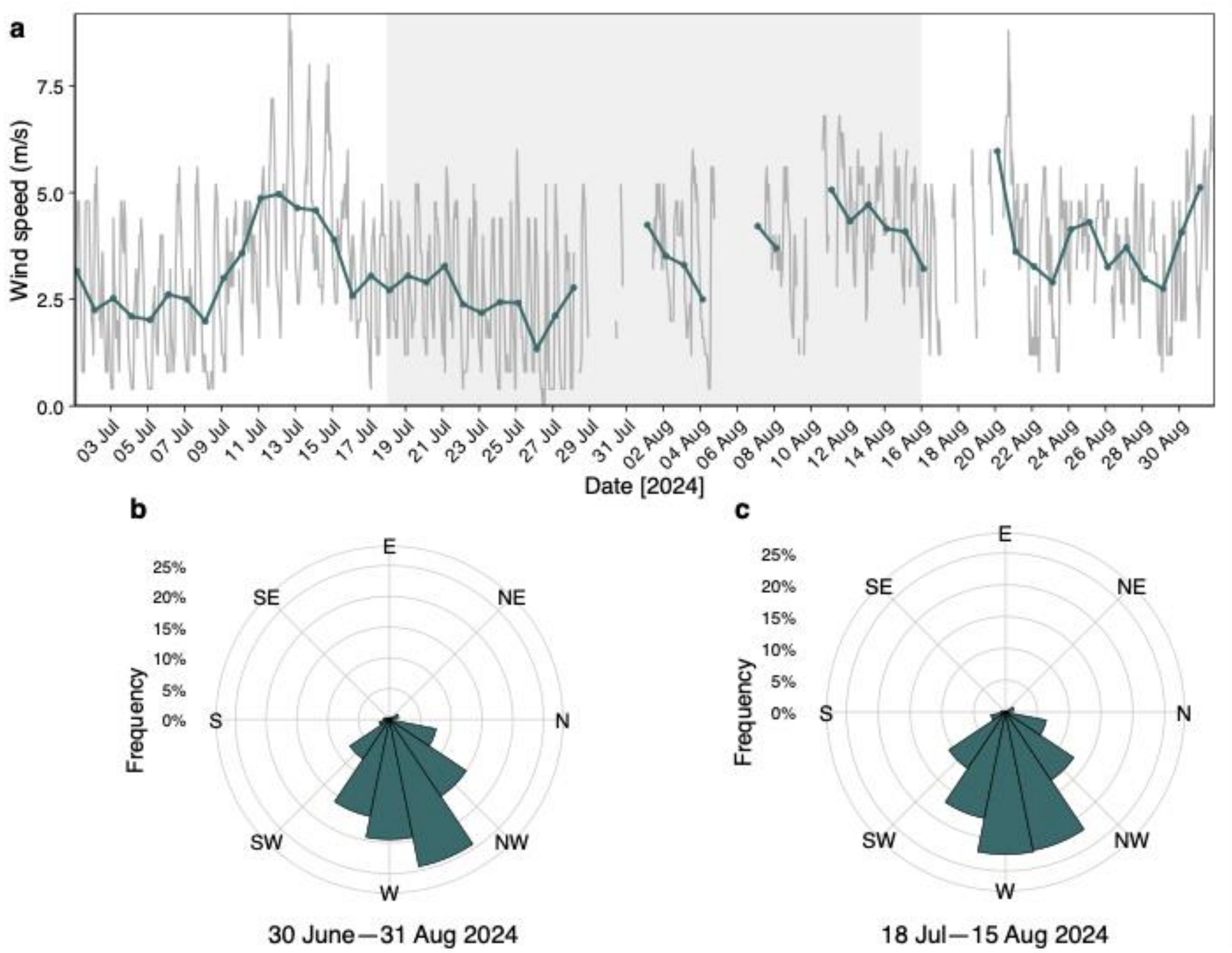

Figure S3 a, Wind speed (m/s) recorded by the SOFAR Spotter buoy at Al Fahal Reef in the central Red Sea from 30 June to 31 August 2024. Raw wind-speed measurements are shown in grey, and the daily mean wind speed is overlaid in green. The shaded period, 27 July–15 August, overlaps with the dates shown in Fig. 2. Daily means are not shown for days with fewer than n = 12 records to avoid bias due to incomplete daily coverage. Panels **b** and **c** show wind-direction rose plots for the full record (30 June to 31 August 2024) and for the highlighted period (18 July to 15 August 2024), respectively.

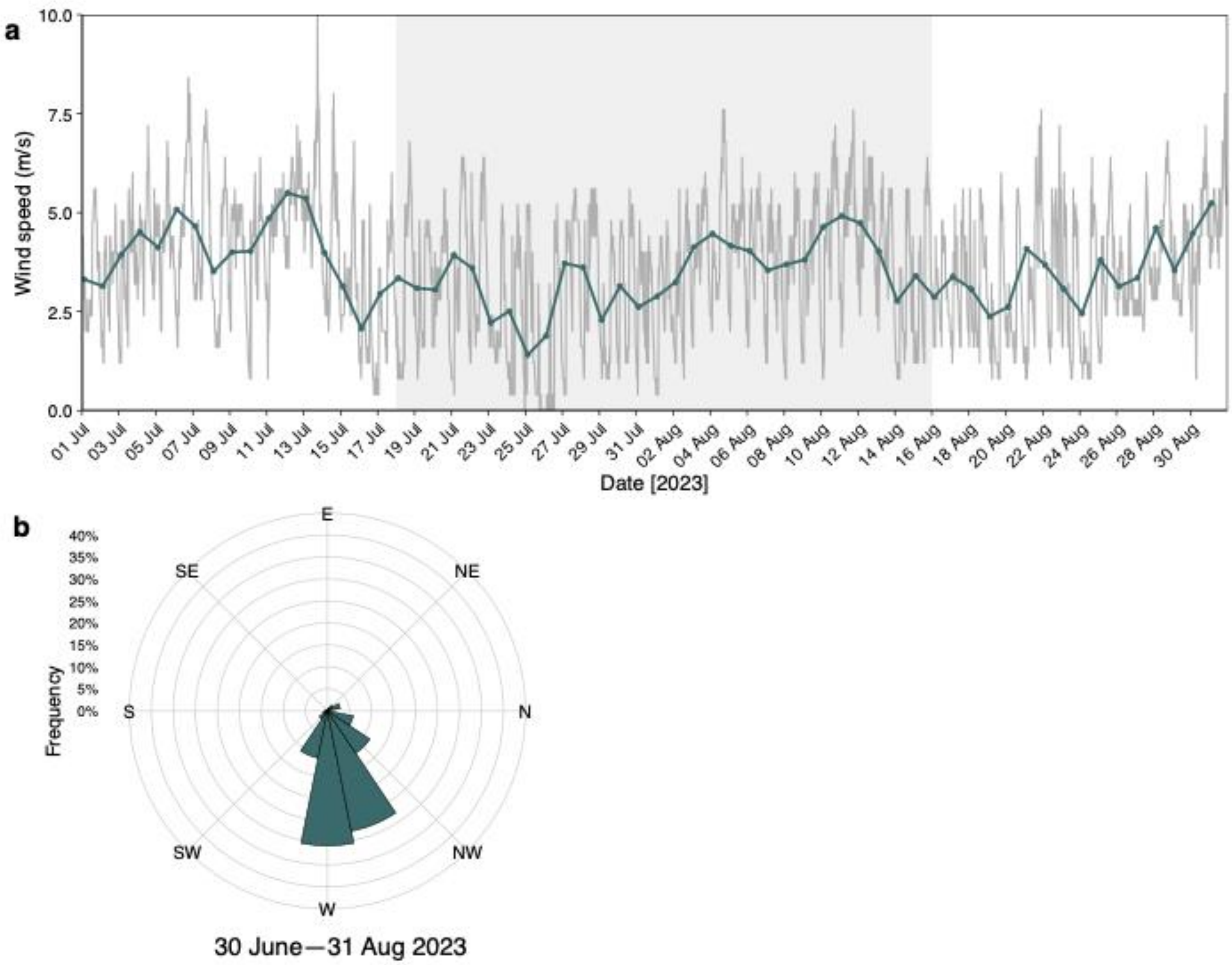

Figure S4 a, Wind speed (m/s) recorded by the SOFAR Spotter buoy at Al Fahal Reef in the central Red Sea from 30 June to 31 August 2023. Raw wind-speed measurements are shown in grey, and the daily mean wind speed is overlaid in green. Panel **b** shows wind-direction rose plots for the full record (30 June to 31 August 2023). Note: y-axis range in panel **a** differs from Fig. S3a.

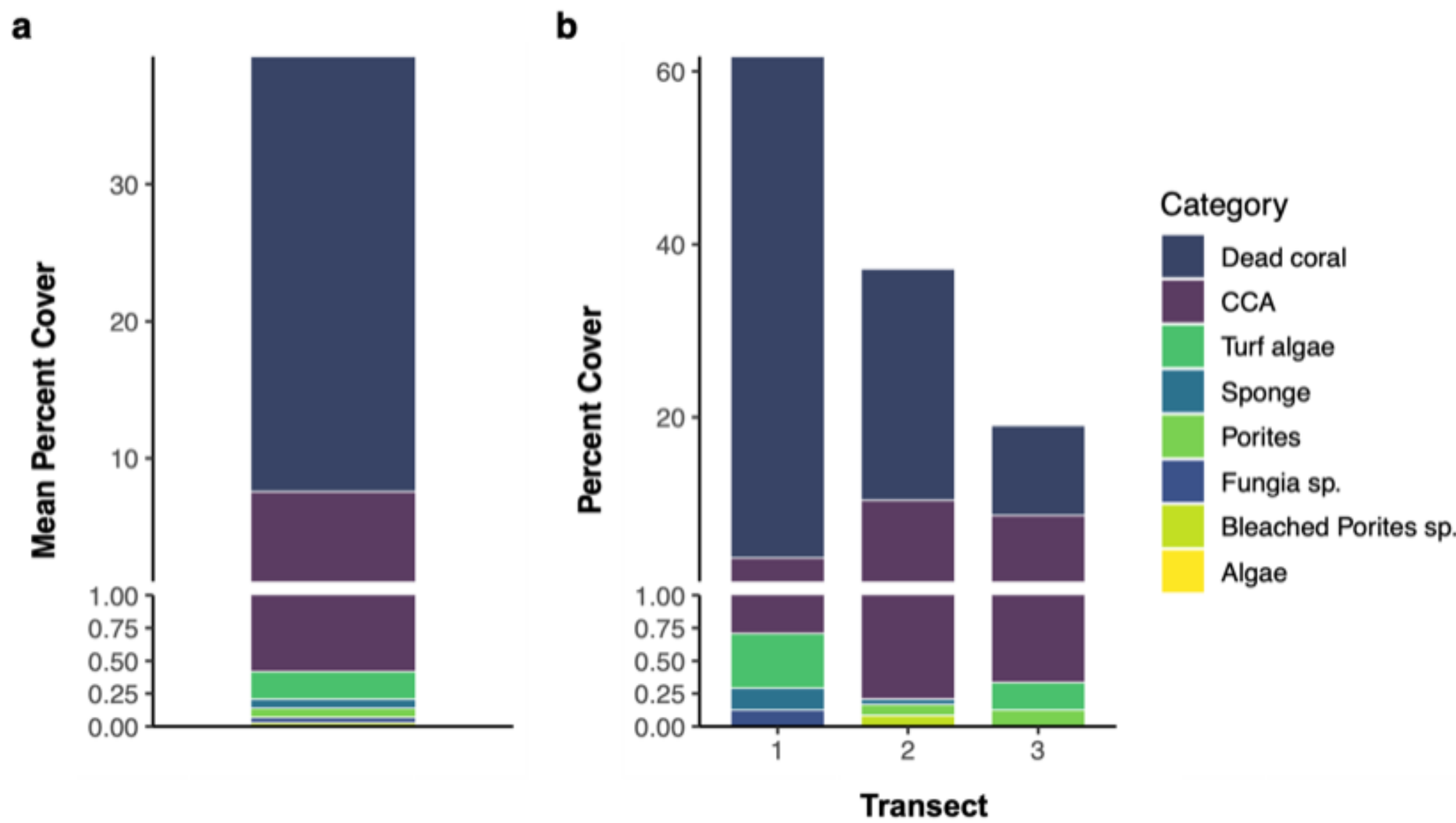

Figure S5 a, Mean percent benthic cover across the three transects in April 2025 on nearby reef habitats, based on CPCe analysis of photo quadrats. **b,** Mean percent cover (%) for each transect separately. **a,b,** Bars are stacked by benthic category, and the y-axis break improves visibility of low-cover categories.

Table S1: Detected marine heatwaves in 2024 at the study site and their characteristics.

Event number	Start date (dd-mm-yyy)	End date	Duration	Max intensity (°C)	Mean intensity (°C)
1	04-04-2024	10-04-2024	7	1.52	1.37
2	19-05-2024	30-05-2024	12	1.55	1.35
3	03-06-2024	18-09-2024	108	2.59	1.65
4	24-09-2024	01-10-2024	8	1.15	0.98
5	10-10-2024	18-10-2024	9	1.13	0.88
6	16-11-2024	23-11-2024	8	1.01	0.87

Table S2. Timing, location, and depth of photo transects surveyed at the study site near the nursery structure location on Al Fahal Reef during post-event surveys.

**Reef site**	**Date (dd/mm/yyyy)**	**Transect ID**	**Latitude**	**Longitude**	**Depth (m)**	**Analysed photos**
Northern Al Fahal (semi-sheltered site)	17/04/2025	AF T1	22°18'23.2"N	38°57'51.2"E	7	N=24
Northern Al Fahal (semi-sheltered site)	17/04/2025	AF T2	22°18'23.2"N	38°57'51.2"E	7	N=24
Northern Al Fahal (semi-sheltered site)	17/04/2025	AF T3	22°18'23.2"N	38°57'51.2"E	7	N=24

Table S3 Images of coral reef taxa in habitats near the coral nursery location after the MHW peak (21 August 2024), showing bleaching and mortality (as evidenced by algal colonisation).

Date & Description	Image
21 August 2024<br><br>Bleached<br><br>*Stylophora pistillata*	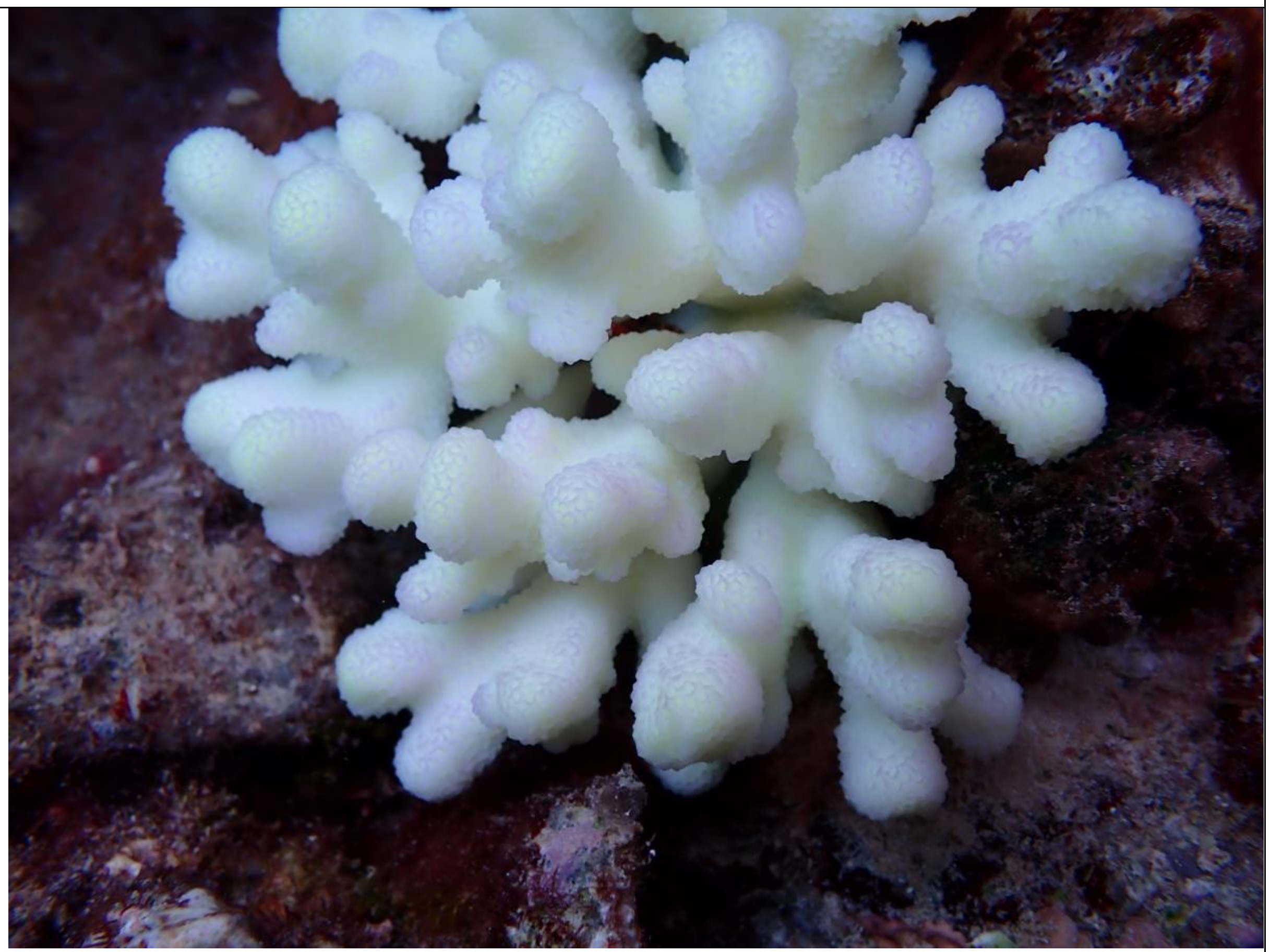

21 August 2024<br><br>Bleached and partial tissue loss<br><br>Pocillopora sp.	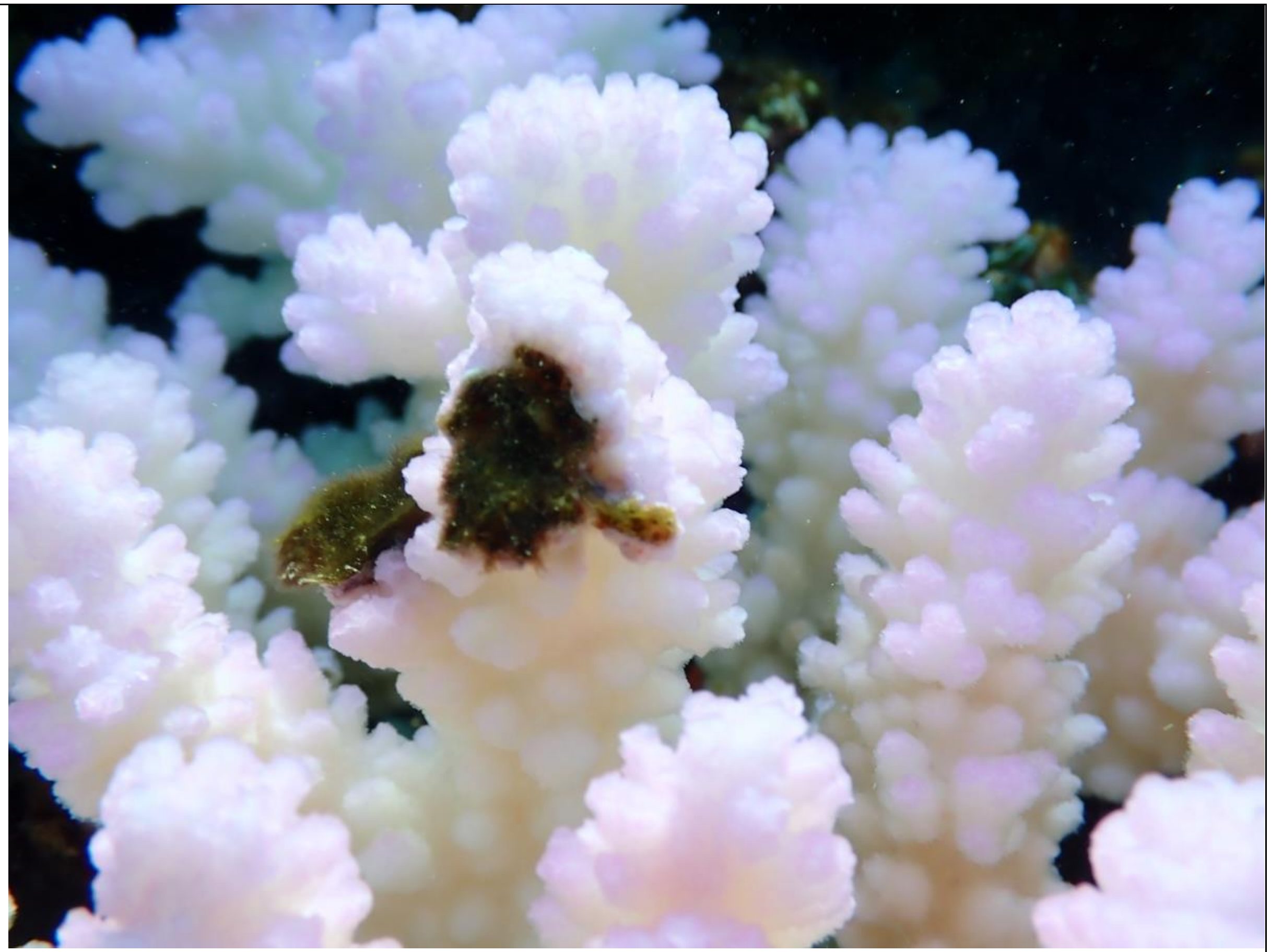

21 August 2024<br><br>Bleached and partially dead, algal overgrowth<br><br>*Stylophora* sp.	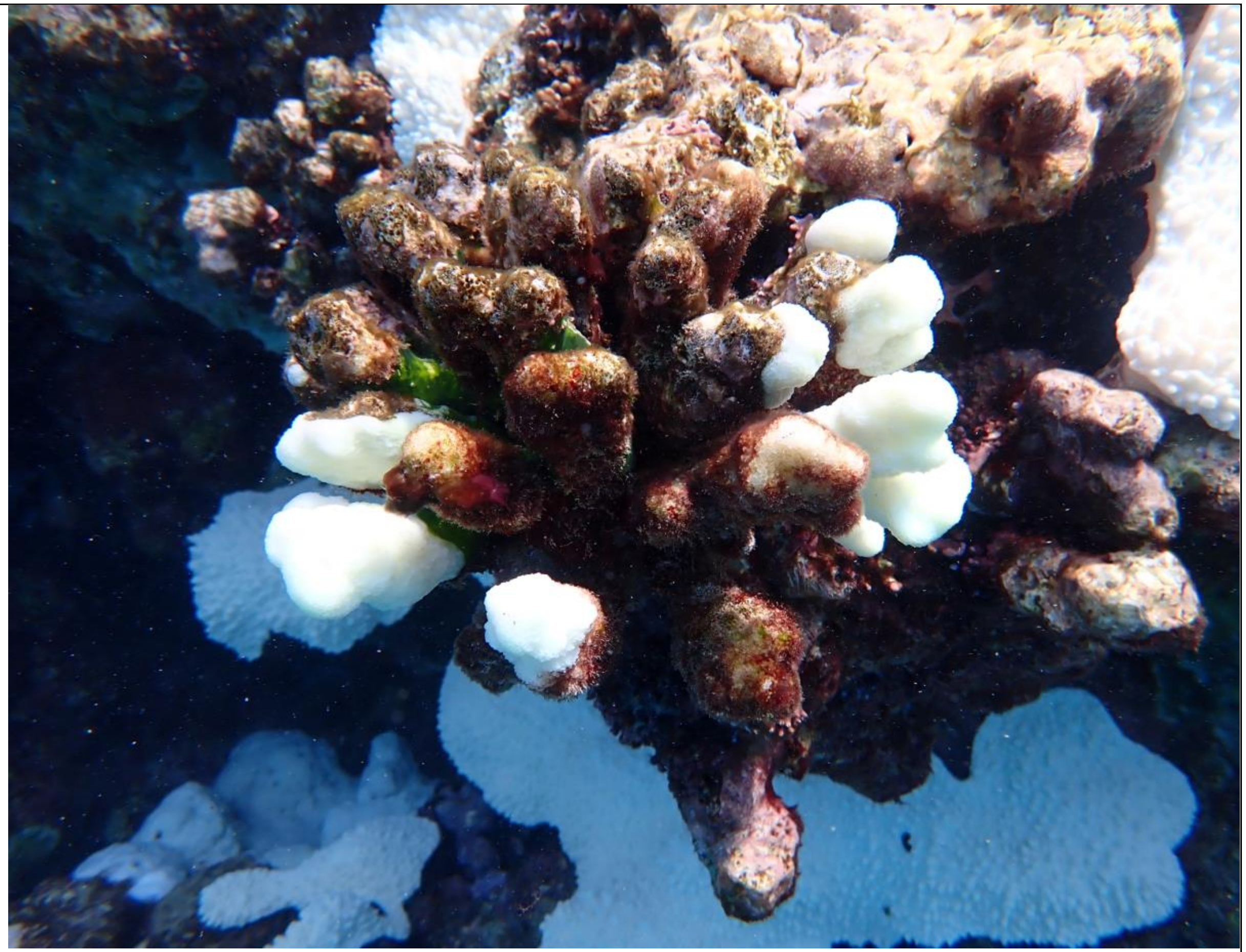

21 August<br><br>Unbleached<br><br>*Lobophyllia sp.*	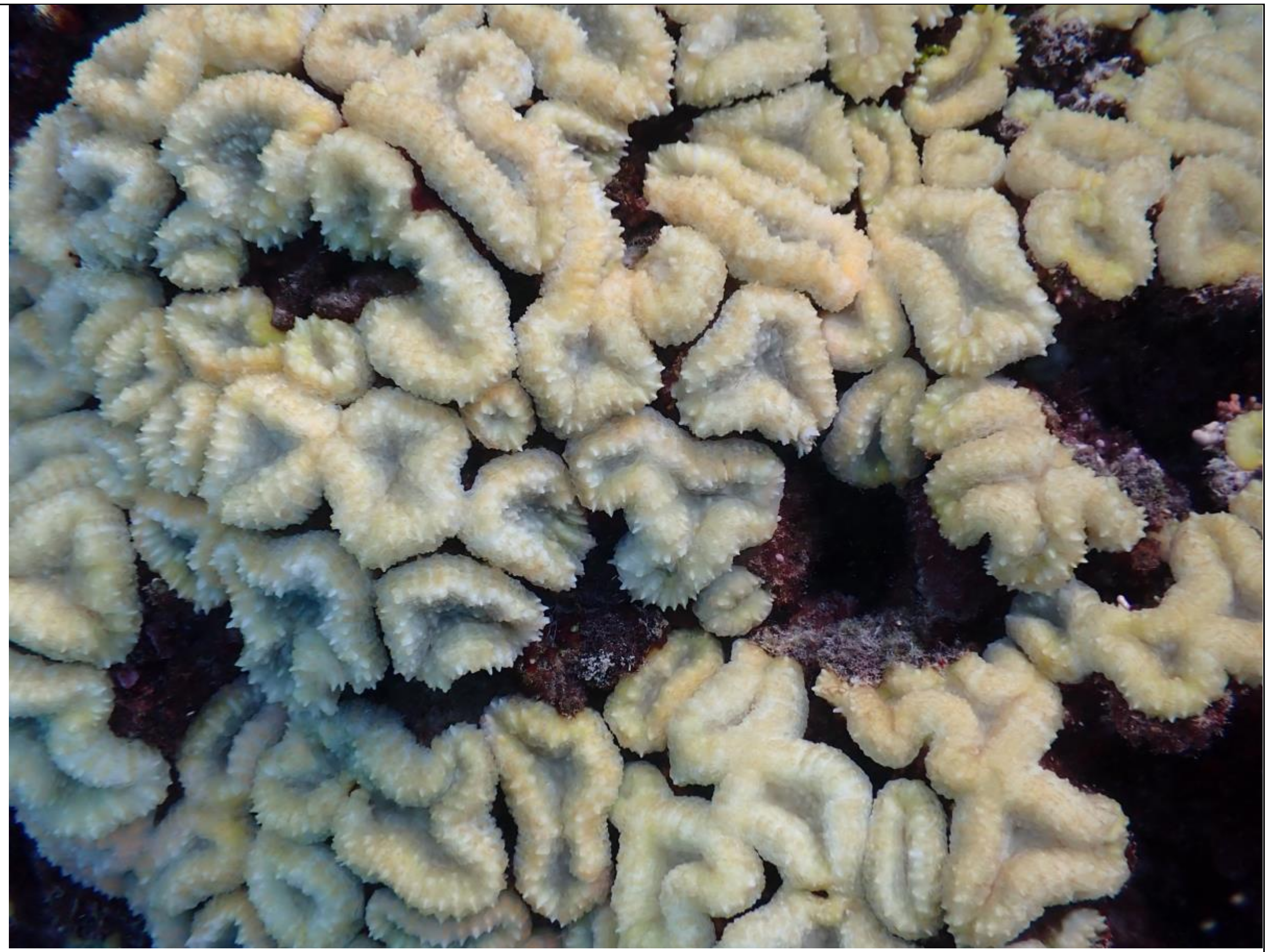

21 August 2024<br><br>Bleached<br><br>*Acropora* sp.	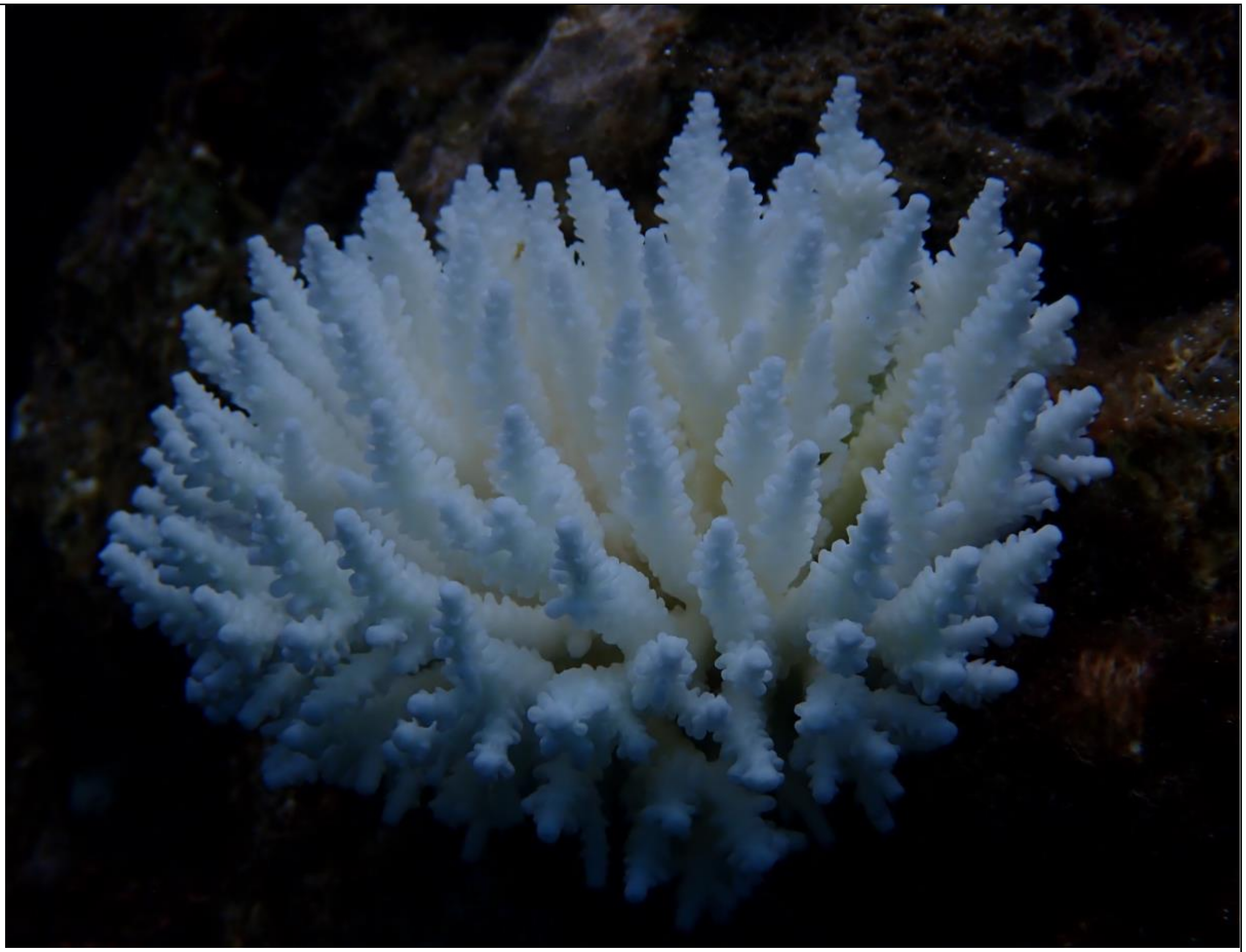

21 August 2024<br><br>Bleached colony with partial mortality at colony centre<br><br>*Acropora* cf. *hemprichii*	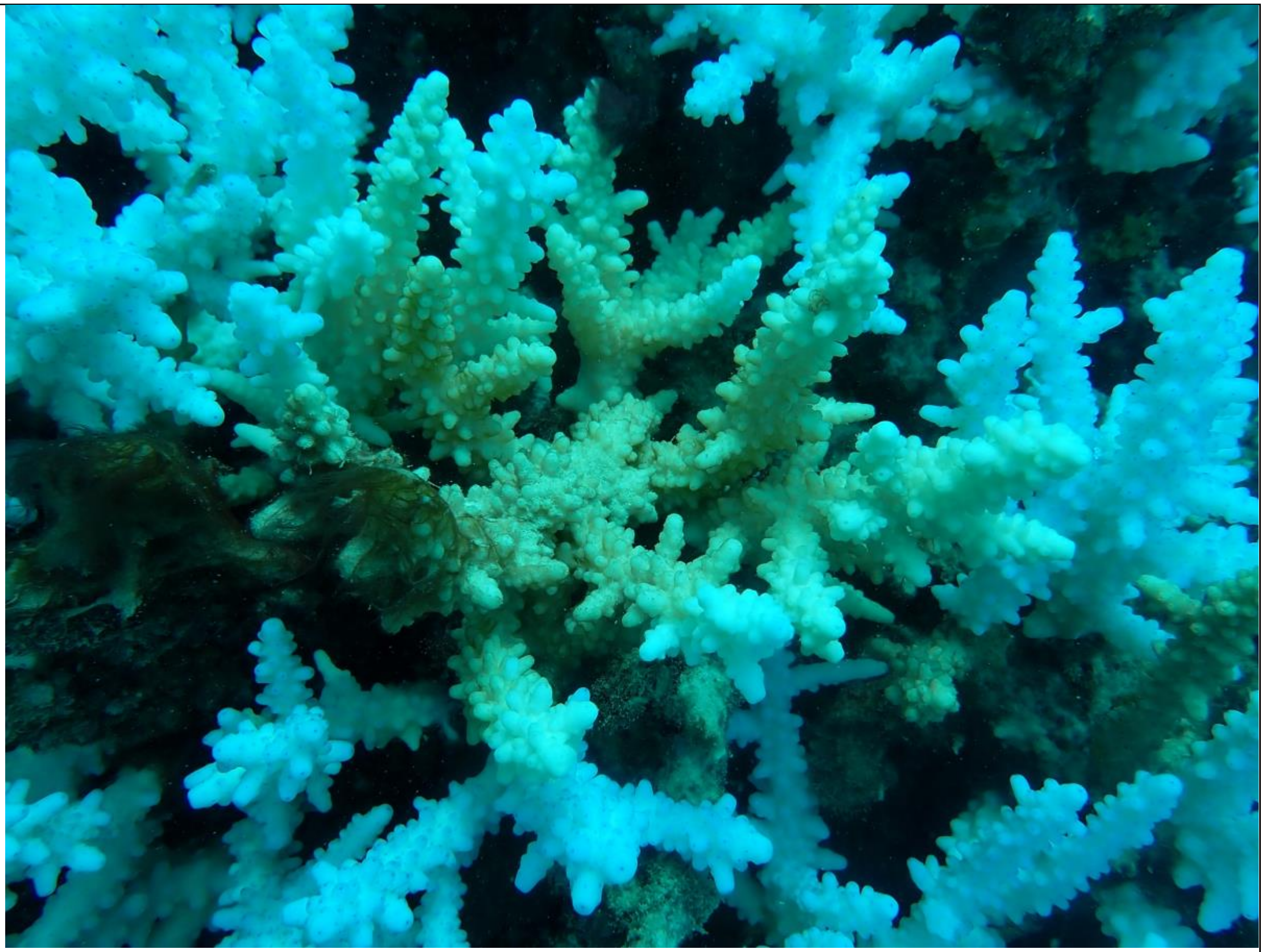

21 August 2024<br><br>Dead colony with signs of algal colonisation<br><br>*Favites* cf. *micropentagonus*	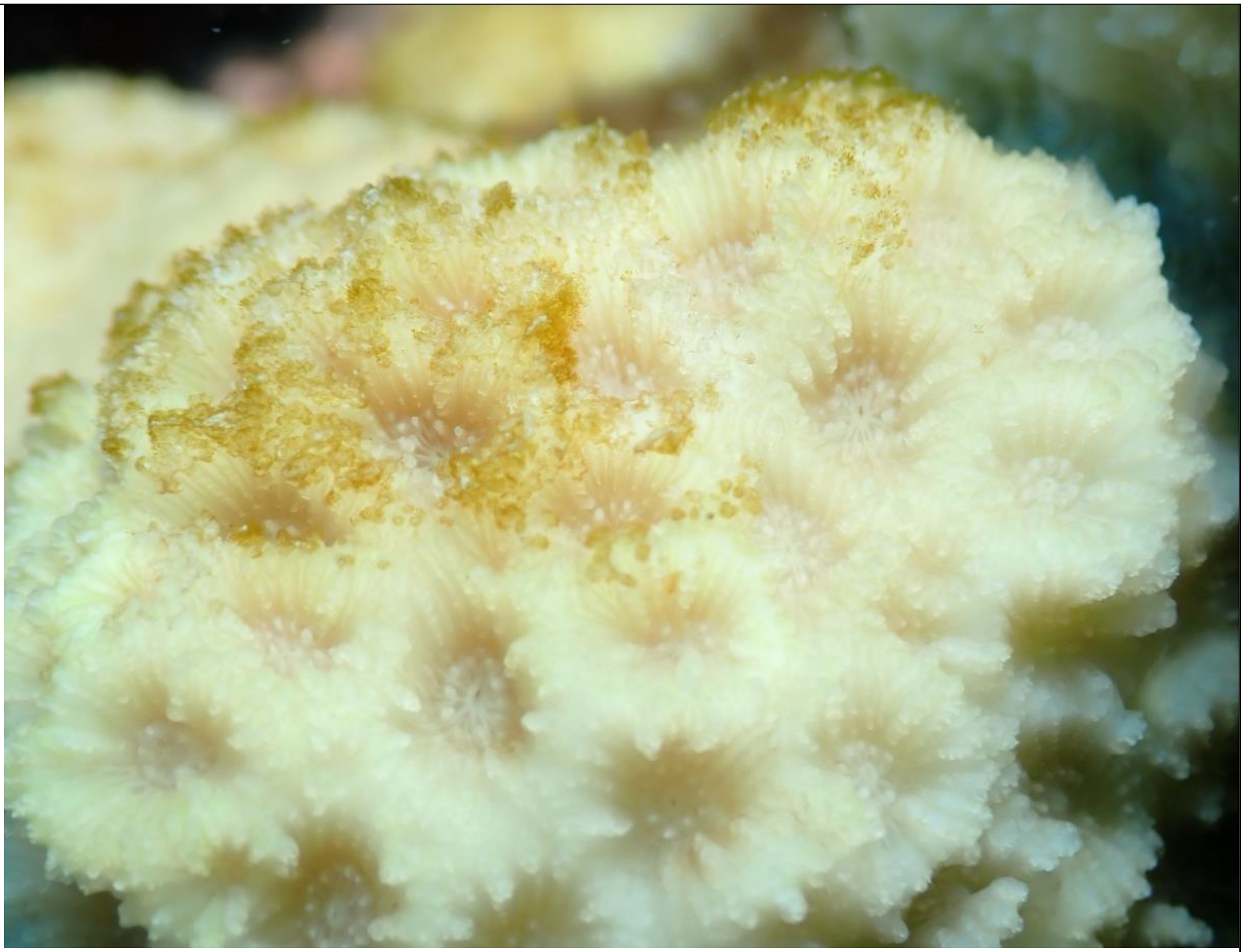

21 August 2024<br><br>Bleached and partial tissue death, with algal colonisation<br><br>*Pocillopora* sp.	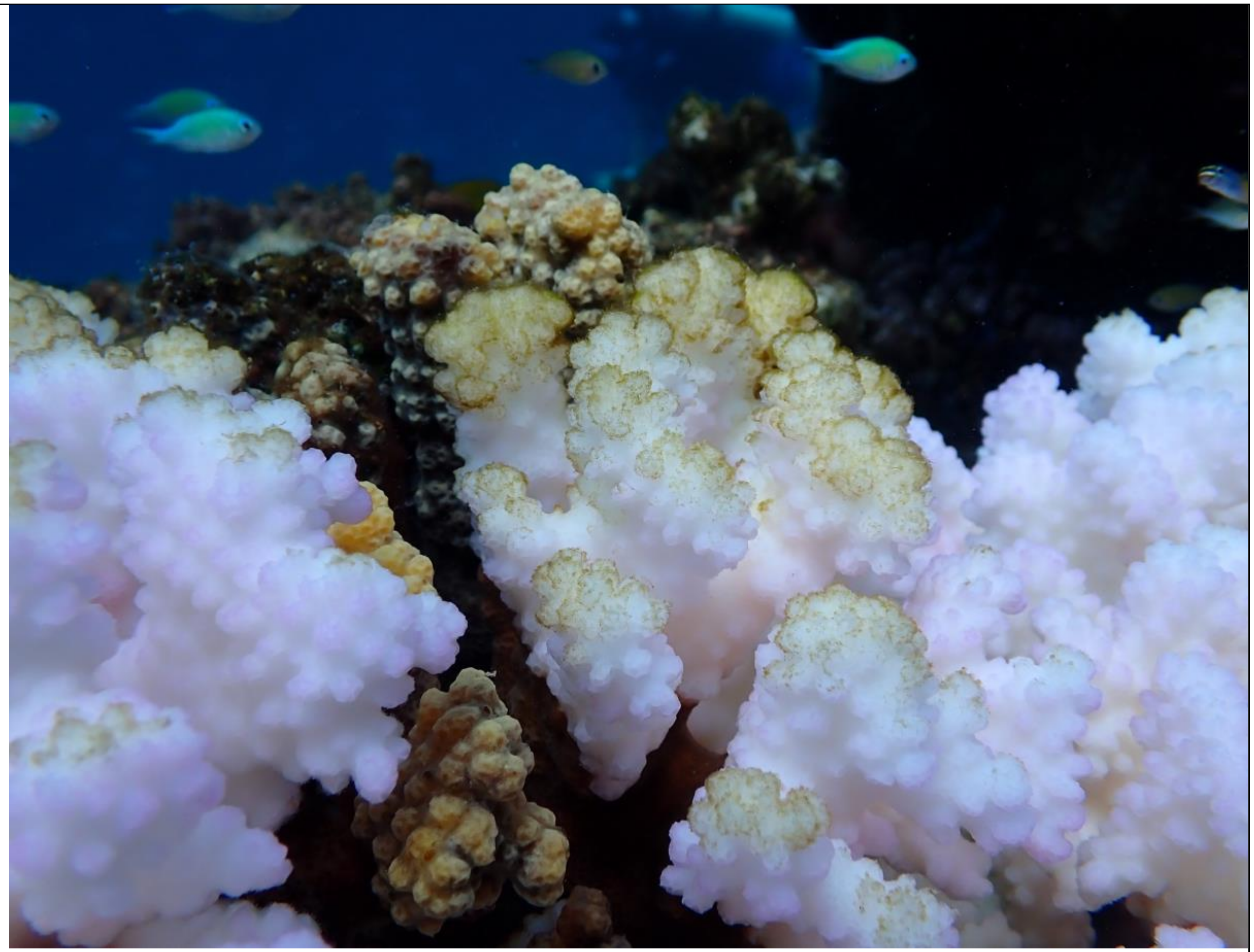

21 August 2024<br><br>Dead colony with algal colonisation<br><br>*Acropora* sp.	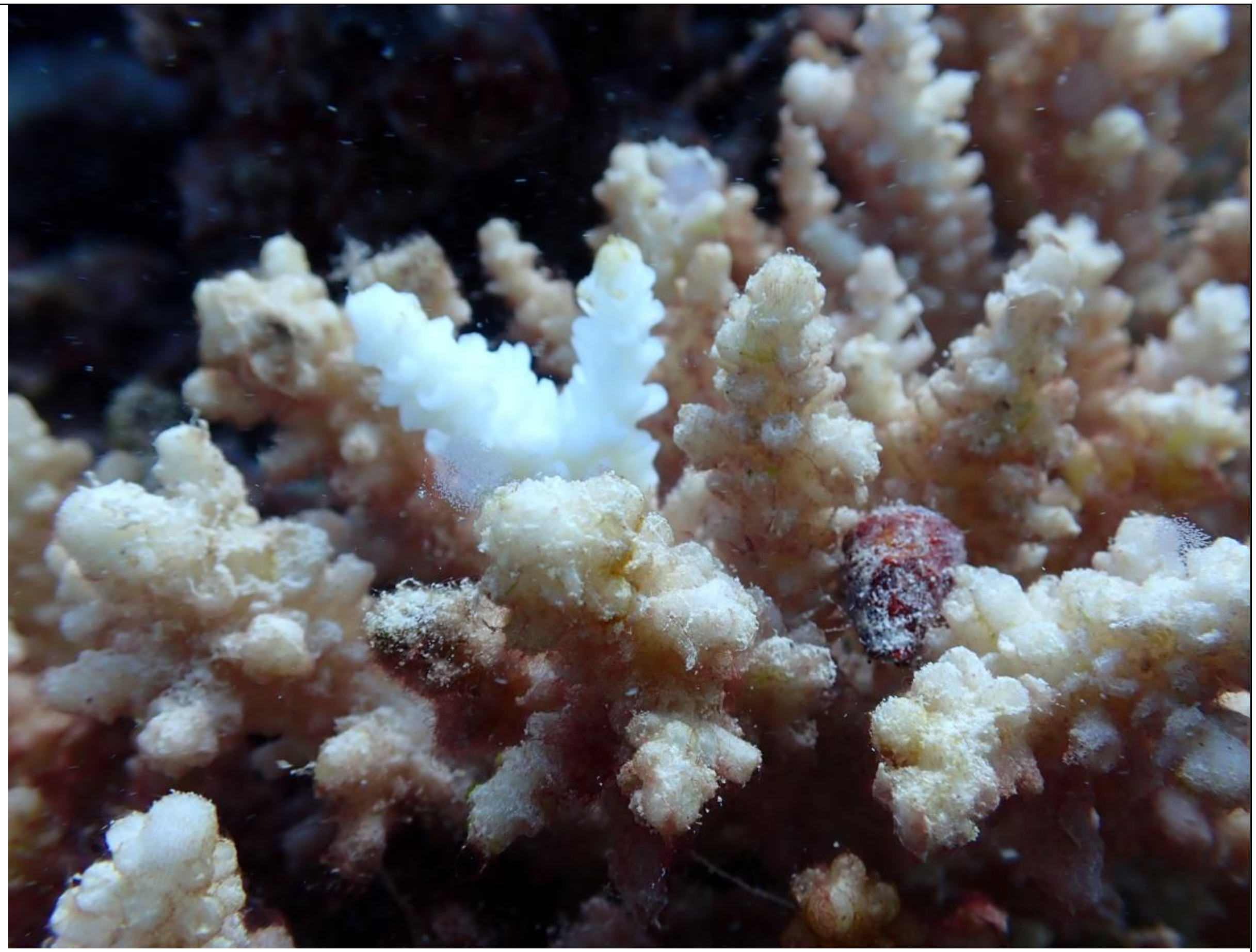

21 August 2024<br><br>Dead colony with algal colonisation<br><br>*Leptastrea* sp.	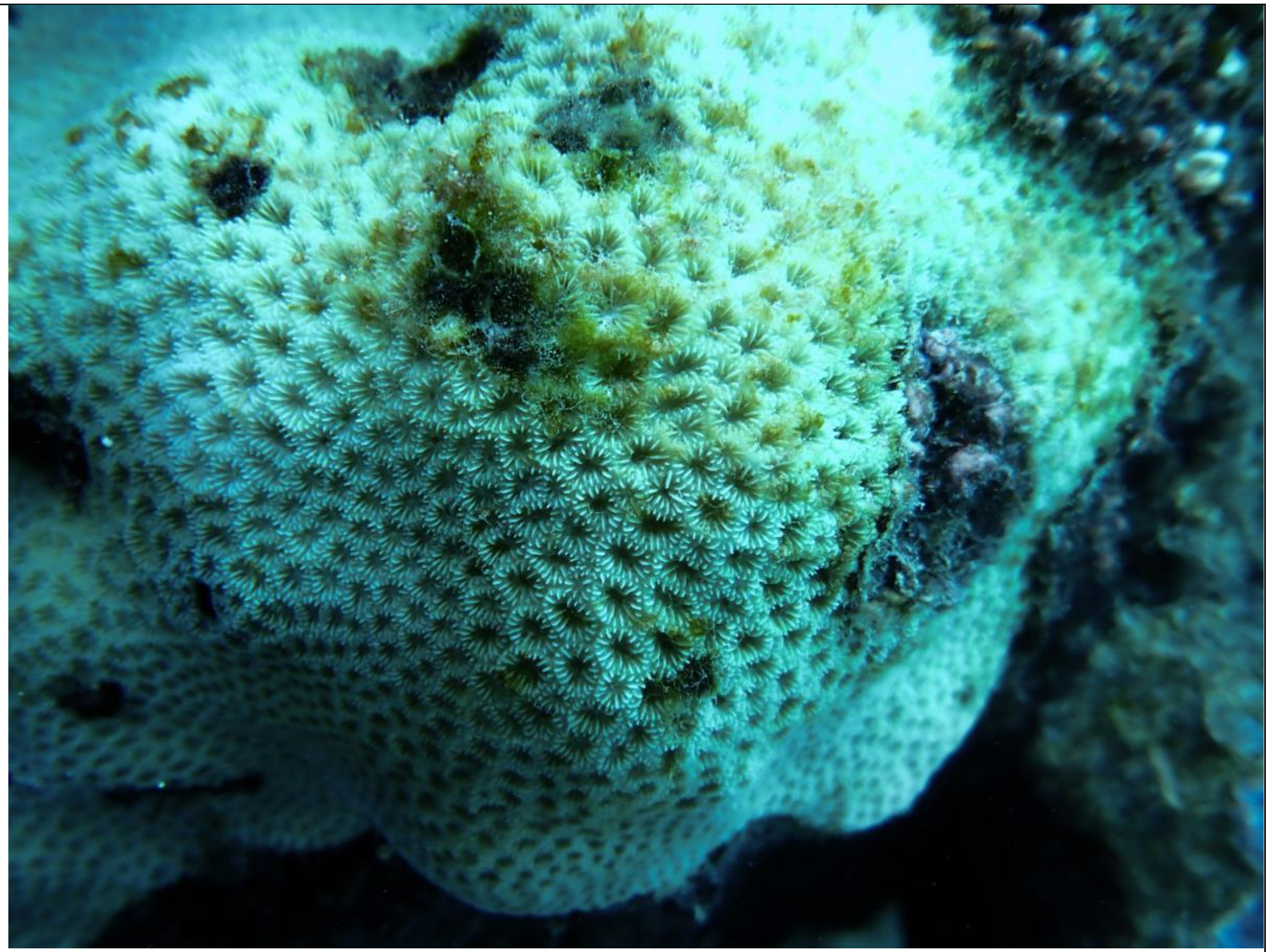

21 August 2024<br><br>Bleached and partial tissue death, with algal growth from the centre<br><br>*Acropora* cf. *hemprichii*	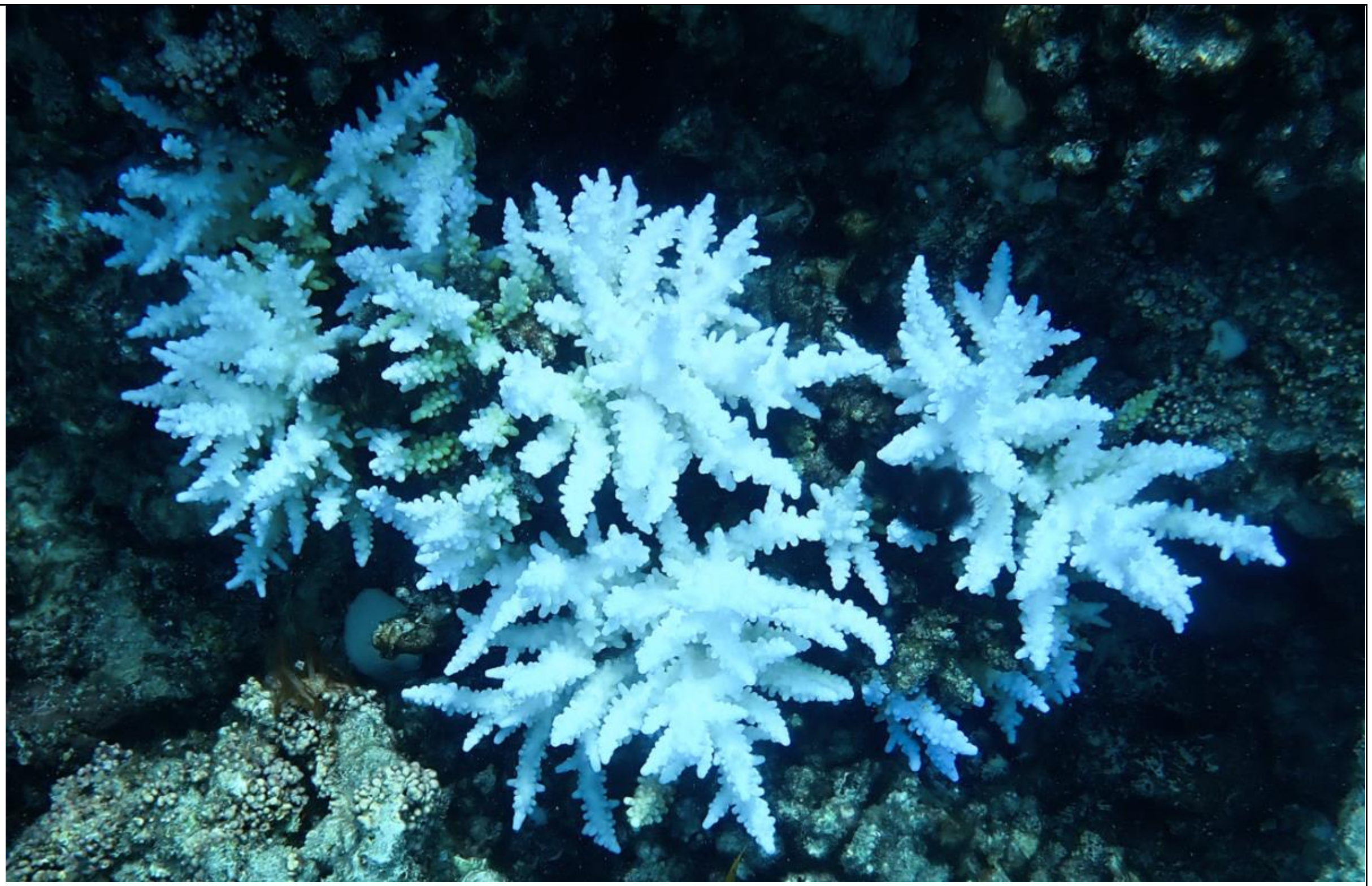

21 August 2024<br><br>Bleached<br>*Tridacna* sp.	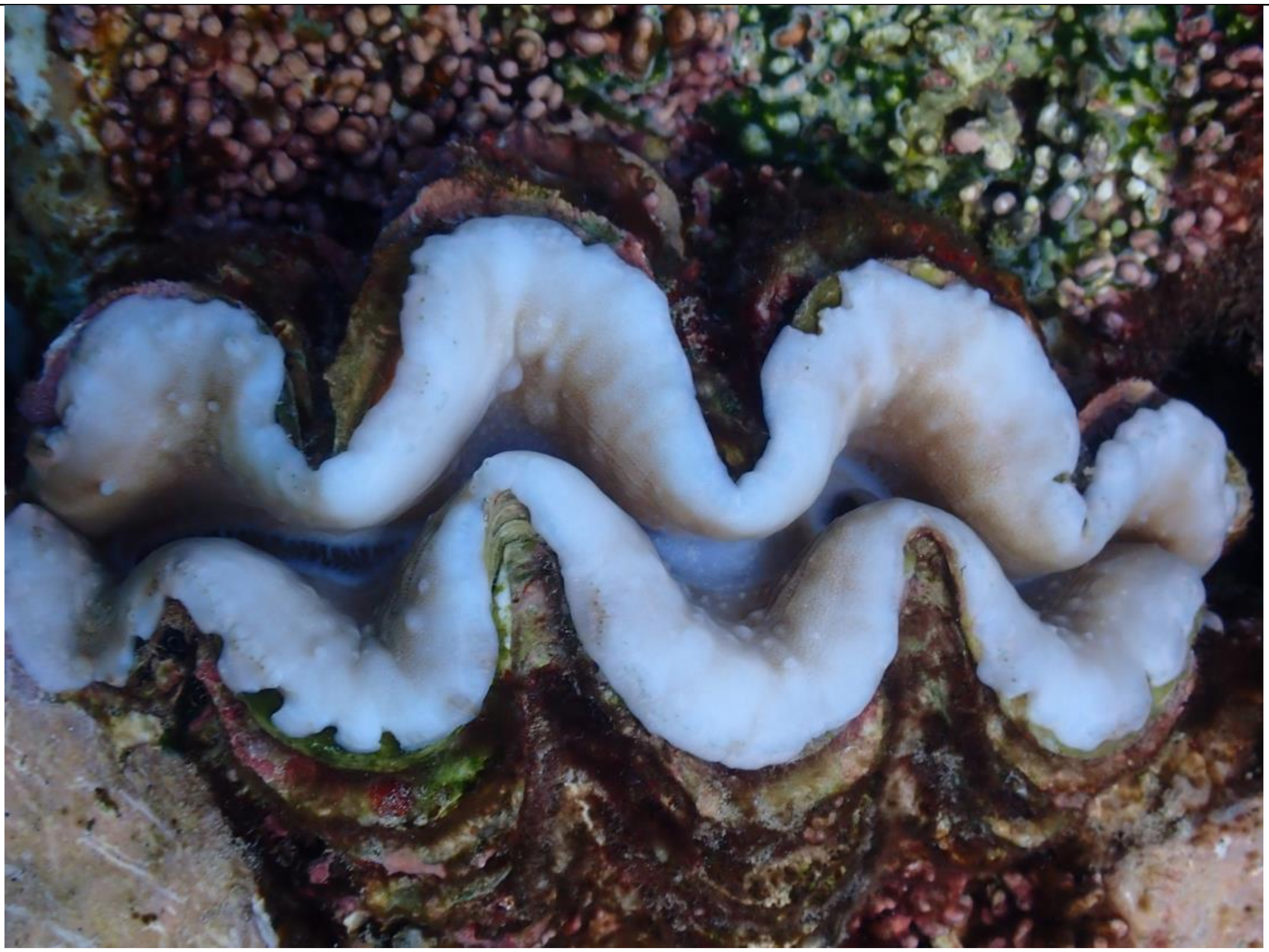

21 August 2024<br><br>Algal colonisation of dead colony<br><br>*Acropora* sp.	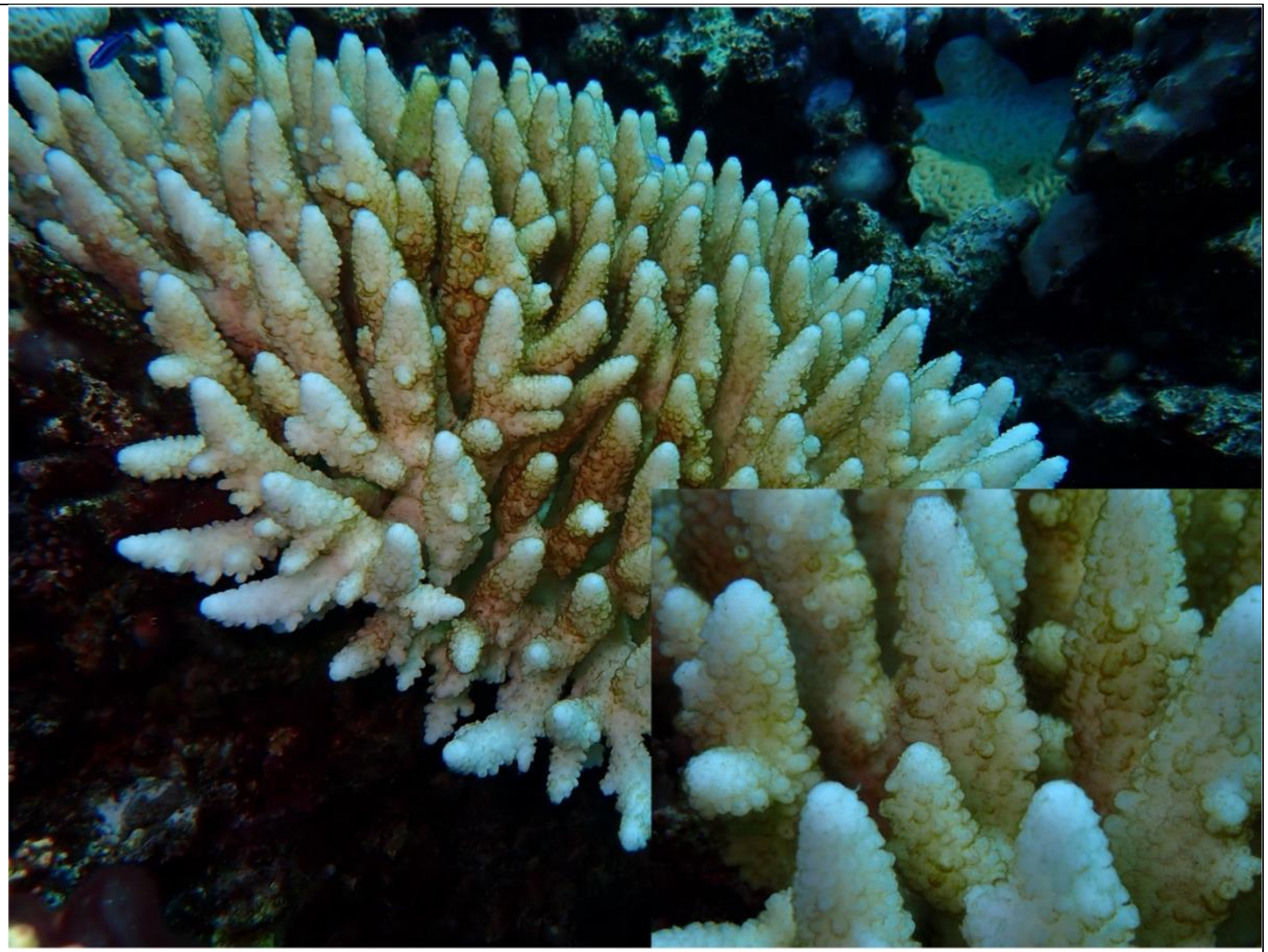

21 August 2024<br><br>Dead colony with algal colonisation<br><br>*Galaxea* cf. *fascicularis*	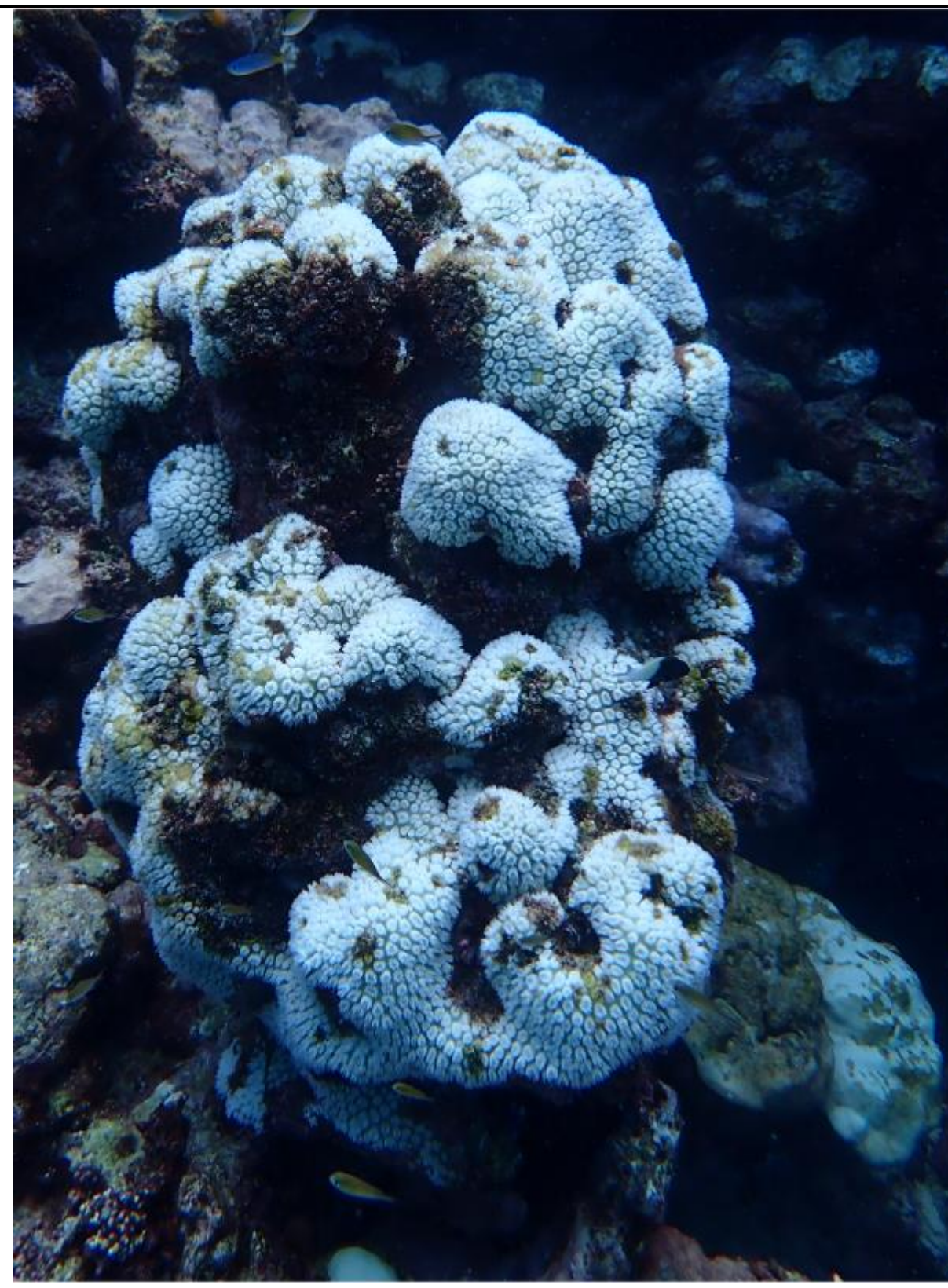 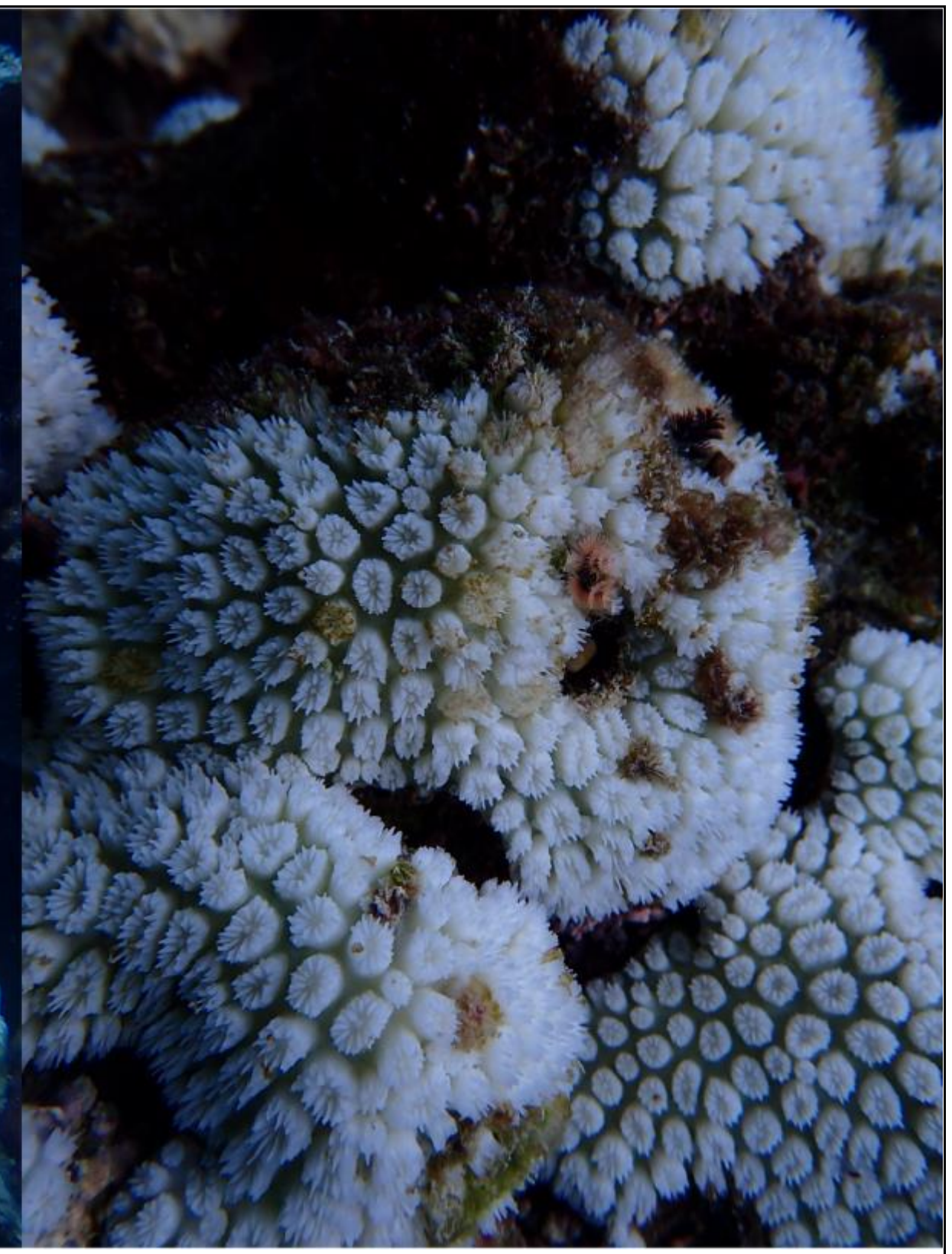

21 August 2024<br><br>General view of sheltered site community, showing bleaching and/or mortality.	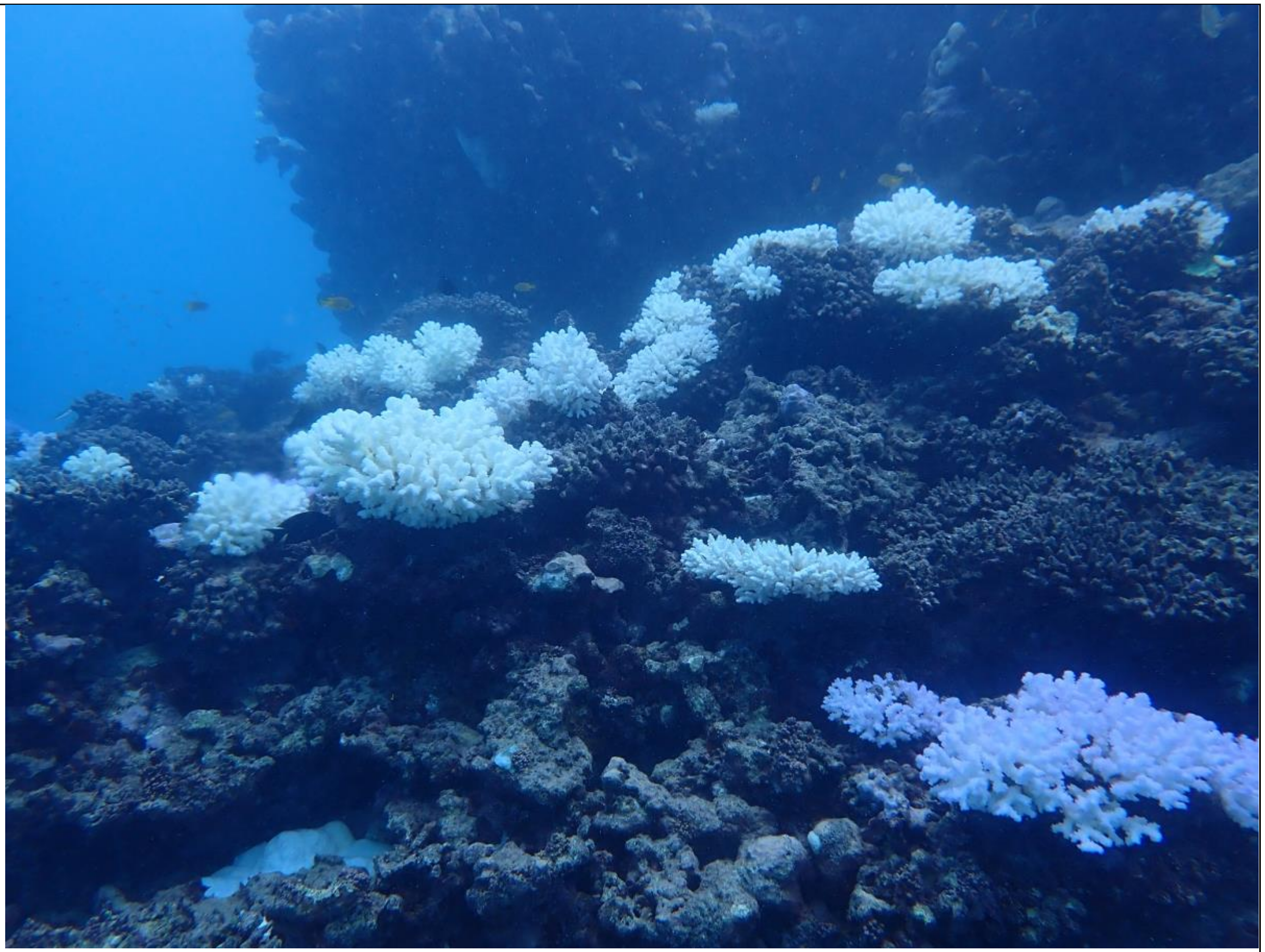

21 August 2024<br><br>General view of study site, showing site-wide impacts.	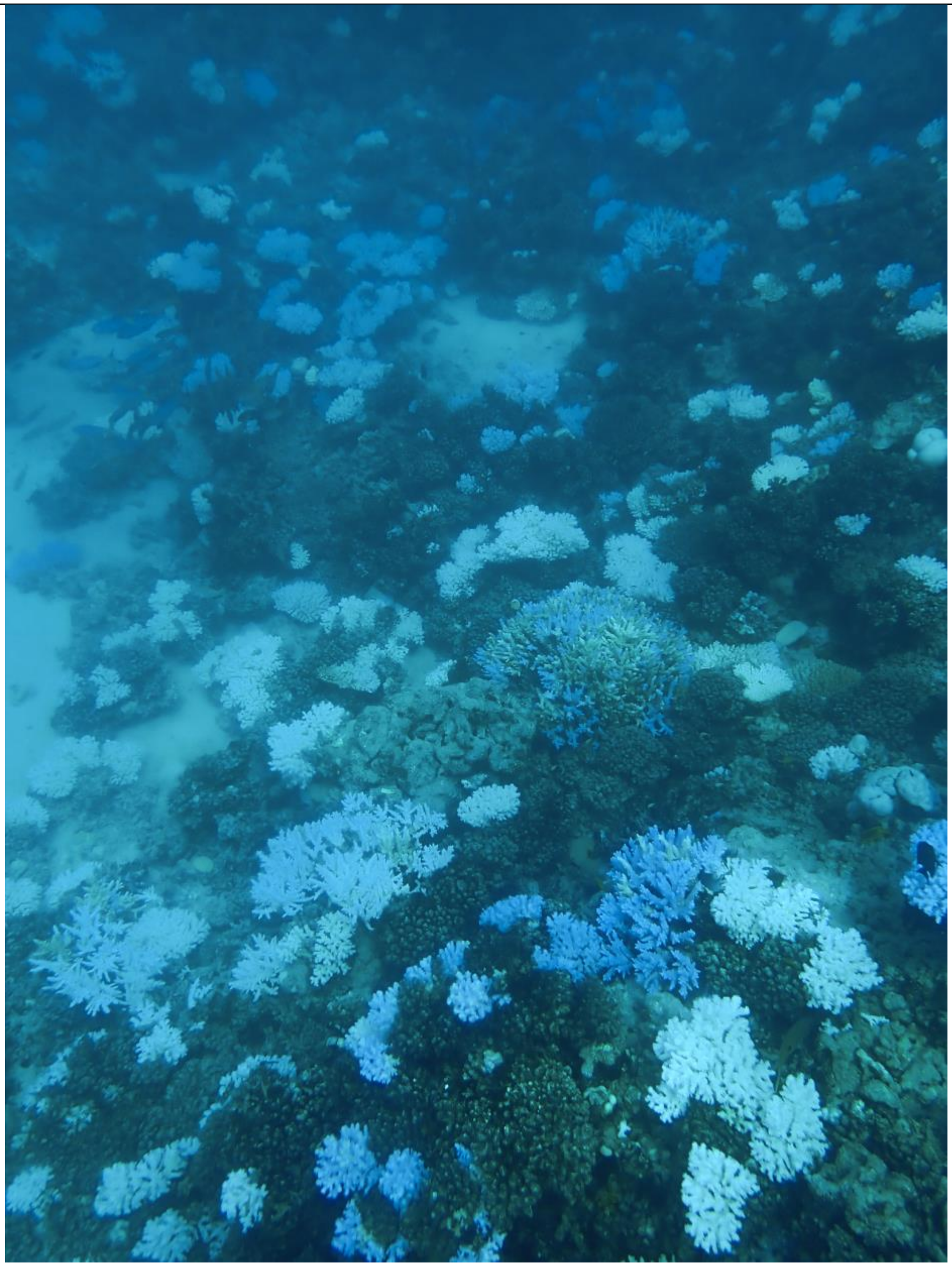

Supplemental References

Pezner, A. K., Courtney, T. A., Barkley, H. C., Chou, W.-C., Chu, H.-C., Clements, S. M., . . . Kline, D. I. (2023). Increasing hypoxia on global coral reefs under ocean warming. *Nature Climate Change, 13*(4), 403-409.
